# Genome-Wide Association Study of Circulating Estradiol, Testosterone, and Sex Hormone-Binding Globulin in Postmenopausal Women

**DOI:** 10.1371/journal.pone.0037815

**Published:** 2012-06-04

**Authors:** Jennifer Prescott, Deborah J. Thompson, Peter Kraft, Stephen J. Chanock, Tina Audley, Judith Brown, Jean Leyland, Elizabeth Folkerd, Deborah Doody, Susan E. Hankinson, David J. Hunter, Kevin B. Jacobs, Mitch Dowsett, David G. Cox, Douglas F. Easton, Immaculata De Vivo

**Affiliations:** 1 Channing Laboratory, Department of Medicine, Brigham and Women’s Hospital and Harvard Medical School, Boston, Massachusetts, United States of America; 2 Department of Epidemiology, Program in Molecular and Genetic Epidemiology, Harvard School of Public Health, Boston, Massachusetts, United States of America; 3 Department of Public Health and Primary Care, University of Cambridge, Cambridge, United Kingdom; 4 Division of Cancer Epidemiology and Genetics, National Cancer Institute, Bethesda, Maryland, United States of America; 5 Academic Department of Biochemistry, Royal Marsden Hospital, London, United Kingdom; 6 Division of Biostatistics and Epidemiology, University of Massachusetts, Amherst, Massachusetts, United States of America; 7 Cancer Research Center of Lyon, INSERM U1052 – CNRS UMR5286, Centre Léon Bérard, Lyon, France; The University of Texas M. D. Anderson Cancer Center, United States of America

## Abstract

Genome-wide association studies (GWAS) have successfully identified common genetic variants that contribute to breast cancer risk. Discovering additional variants has become difficult, as power to detect variants of weaker effect with present sample sizes is limited. An alternative approach is to look for variants associated with quantitative traits that in turn affect disease risk. As exposure to high circulating estradiol and testosterone, and low sex hormone-binding globulin (SHBG) levels is implicated in breast cancer etiology, we conducted GWAS analyses of plasma estradiol, testosterone, and SHBG to identify new susceptibility alleles. Cancer Genetic Markers of Susceptibility (CGEMS) data from the Nurses’ Health Study (NHS), and Sisters in Breast Cancer Screening data were used to carry out primary meta-analyses among ∼1600 postmenopausal women who were not taking postmenopausal hormones at blood draw. We observed a genome-wide significant association between SHBG levels and rs727428 (joint β = -0.126; joint P = 2.09×10^–16^), downstream of the *SHBG* gene. No genome-wide significant associations were observed with estradiol or testosterone levels. Among variants that were suggestively associated with estradiol (P<10^–5^), several were located at the *CYP19A1* gene locus. Overall results were similar in secondary meta-analyses that included ∼900 NHS current postmenopausal hormone users. No variant associated with estradiol, testosterone, or SHBG at P<10^–5^ was associated with postmenopausal breast cancer risk among CGEMS participants. Our results suggest that the small magnitude of difference in hormone levels associated with common genetic variants is likely insufficient to detectably contribute to breast cancer risk.

## Introduction

A family history of breast cancer is one of the strongest known risk factors for the disease, with ∼2-fold increased risk in first degree relatives of cases [1]. Twin studies suggest that around a quarter of the variance in breast cancer risk is due to inherited genetic factors [2]. The *BRCA1* and *BRCA2* genes are both associated with high risks of breast and ovarian cancer. However, these mutations are rare, accounting for only 2–4% of all breast cancers in non-founder populations [3] and about 20% of excess familial risk [4]. Among non-*BRCA* mutation carriers, genetic susceptibility to breast cancer follows a polygenic model where a large number of variants each confer a small effect on risk [5]. Within the last 5 years, the agnostic approach of using genome-wide association studies (GWAS) has successfully identified several additional genetic risk variants. Together, the newly identified alleles account for 7–8% of variation in familial risk [6], suggesting that further variants exist. As yet unidentified variants will likely have frequencies and/or effect sizes such that they will be hard to detect by GWAS using sample sizes that are presently realistic. One alternative approach is to improve power by looking for variants associated with quantitative traits that in turn affect disease risk.

Sex steroid hormones play a primary role in postmenopausal breast cancer etiology [7]. Endogenous and exogenous characteristics that modify exposure to steroid hormones are among the established risk factors for breast cancer development, including reproductive history prior to menopause [8], adiposity after menopause [9], oral contraceptive use [10] and post-menopausal hormone replacement therapy [11]. Additionally, direct measurement of circulating hormone levels of estradiol and testosterone [12] have shown higher levels to be strongly and consistently associated with increased breast cancer risk. Conversely, risk may be inversely related to plasma levels of sex hormone-binding globulin (SHBG) [13]–[15], the major sex steroid transport protein that binds estradiol and testosterone with high affinity, which may limit their bioavailability.

Evidence of heritability has been observed for plasma levels of estradiol, testosterone, and SHBG. Family and twin studies among women estimate genetic factors contribute between 0–45% of variation in log estradiol levels [16], 12–66% of variation in log testosterone levels [16], [17], and 29–83% of variation in log SHBG levels [16], [17]. Well-established associations exist between variants within the *SHBG* gene (Gene ID: 6462) and circulating SHBG levels [18]–[24] and between *CYP19A1* variants and levels of estradiol in post-menopausal women [20], [22], [25], while two variants within the *SHBG* gene region and an X chromosome SNP have recently been reported to be associated with testosterone concentrations in men [24]. Even so, a comprehensive assessment of common variants within known sex steroid hormone synthesis and metabolism pathway genes (i.e. *CYP17A1*, Gene ID: 1586; *CYP19A1*, Gene ID: 1588) found no significant associations with breast cancer risk [26]. Given that genes regulating these complex hormonal pathways are not fully understood or described, variants in genes that are not yet characterized and/or hypothesized to influence hormone synthesis and metabolism *a priori* may be associated with sex hormone levels and thus breast cancer risk.

To describe such novel genotype-phenotype associations we conducted a GWAS of postmenopausal estradiol, testosterone, and SHBG plasma levels. Our study included a subset of women from the National Cancer Institute Cancer Genetic Markers of Susceptibility (CGEMS) project and the GWAS carried out in the framework of the Sisters in Breast Screening (SIBS) study for whom circulating steroid hormones had been measured. Identifying genetic determinants of hormonal levels may provide new insights into breast cancer etiology.

## Results

Characteristics of each study population are shown in **Table 1**. All NHS and SIBS study participants were postmenopausal at the time of blood collection. Among women who were not on PMH at time of blood draw, a greater percentage of NHS participants had never used PMH compared to women in the SIBS study. On average, SIBS participants were slightly older and had a greater BMI at blood draw compared to women in the NHS. Mean testosterone and SHBG levels were very similar between SIBS participants and NHS non-PMH users, although mean estradiol levels were lower among the SIBS women. It is known that measurements of hormone concentrations are prone to substantial variability between different laboratories, because of the different assays used [13]. NHS PMH users had ∼3-fold higher mean estradiol levels and ∼2-fold higher mean SHBG levels compared to NHS women who were not on PMH. Mean testosterone levels were comparable between the PMH and non-PMH groups. No evidence of systematic bias was observed in the GWAS meta-analyses of the three hormones (genomic inflation factor λ ≤1.02 for each; **Figures S1, S2, S3**).

**Table 1:** Select characteristics of study populations.

	Nurses' Health Study		Sisters in Breast Screening Study
	*Non-PMH users*(N = 804)	*PMH users*(N = 899)	(N = 819)
Never PMH use	64%	–	51%
Past PMH use	36%	–	49%
Mean age (SD)	61.4 (4.8)	59.9 (5.4)	62.1 (5.4)
Mean age at menopause (SD)	49.9 (4.0)	48.8 (5.0)	48.9 (5.8)
Mean BMI (kg/m^2^; SD)	26.4 (5.0)	24.7 (4.2)	27.3 (5.3)
E2 measurements (N)	779	668	804
Mean E2 (pmol/L; SD)^a^	29.2 (19.6)	87.1 (79.1)	20.3 (22.9)
T measurements (N)	770	875	819
Mean T (nmol/L; SD)^a^	0.82 (0.5)	0.85 (0.5)	0.82 (0.5)
SHBG measurements (N)	779	898	819
Mean SHBG (nmol/L; SD)	55.1 (29.0)	112.5 (66.2)	55.0 (26.2)

a Corresponding mean E2 concentrations in pg/ml are 8.0, 23.7, and 5.5 and mean T concentrations in ng/dl are 23.7, 24.5, and 23.7 for NHS non-PMH users, NHS PMH users, and SIBS participants, respectively.

### Sex Hormone-binding Globulin

Genome-wide significant SNPs (P<5×10^–8^) associated with SHBG levels were observed on chromosome 17 (**Table 2** **, Figure S4**). The most significant association with SHBG levels was observed for rs727428, which resides ∼1.1 kb from the 3′ end of the *SHBG* gene, in both the NHS non-PMH user (P = 4.08×10^–8^) and SIBS (P = 8.27×10^–10^) study populations (**Table S1**). The joint P-value for the association between rs727428 and SHBG levels was P = 2.09×10^–16^. Effect estimates did not display between-study heterogeneity (P_heterogeneity_ = 0.59; **Table 2**). Sixteen additional SNPs that span ∼110 kb across the region on chromosome 17p13 that includes the *SHBG* gene reached genome-wide significance (P≤1.93×10^–8^). No significant between-study heterogeneity was observed for these SNPs (P_heterogeneity_≥0.19; **Table S1**). No SNP achieved genome-wide significance among the group of NHS women who were on PMH at the time of blood draw (P≥1.07×10^–6^). Weaker effect estimates for SNP associations with SHBG levels were observed at the *SHBG* locus among PMH users, resulting in some moderate between-study heterogeneity in secondary meta-analyses of the 3 groups (I^2^≤66%, P_heterogeneity_≥0.05; **Table S2)**.

**Table 2:** Summary of regions associated with log SHBG, log estradiol, and log testosterone levels at P<10^−5^ from GWAS meta-analyses. of non-PMH users.

Hormone	Region^a^	no.snps	span(kB)	min-maxposition (bp)^a^	best SNP	alleles^b^	MAF^c^	β^d^	P-value^d^	P_heterogeneity_ d	Genes within20kb of bestSNP^a^
SHBG	17p13	29	261	7259120–7520197	rs727428	C/T	0.42	-0.126	2.09E-16	0.59	FXR2/SHBG/SAT2/ATP1B2
SHBG	5q14	4	75	89070647–89145506	rs10514317	C/T	0.12	-0.132	6.96E-07	0.13	
SHBG	2q37	1	-	241732719	rs6721345	G/A	0.01	1.130	2.60E-06	0.35	SNED1/MTERFD2
SHBG	1q23	2	3	160688161–160690928	rs424950	G/C	0.48	0.076	4.76E-06	0.43	
SHBG	17q23	9	18	53163063–53181345	rs8077059	T/C	0.25	-0.081	5.40E-06	0.45	
SHBG	3p12	1	-	76566873	rs3849491	C/T	0.48	-0.073	5.52E-06	0.93	
SHBG	10p15	5	5	4135640–4141121	rs10795130	T/G	0.13	0.103	6.97E-06	0.48	
SHBG	16q12	1	-	52585472	rs12596210	T/C	0.12	-0.120	8.74E-06	0.48	FTO
estradiol	20q12	11	127	37725829–37853256	rs6016142	C/T	0.11	-0.179	6.47E-08	0.23	
estradiol	15q21	34	66	49285831–49351633	rs727479	A/C	0.34	-0.107	5.11E-07	0.25	CYP19A1/MIR4713
estradiol	2p23	11	28	31373046–31401477	rs597800	G/C	0.15	-0.148	5.29E-07	0.84	EHD3
estradiol	7p15	8	94	29788672–29882593	rs10488084	A/C	0.08	0.180	2.37E-06	0.76	FKBP14/PLEKHA8
estradiol	11q12	1	-	61468707	rs2727261	C/T	0.09	0.159	3.27E-06	0.73	BEST1/FTH1
estradiol	19q12	2	17	36585958–36602969	rs11880316	C/A	0.01	0.414	3.63E-06	0.73	
estradiol	18q22	1	-	70916034	rs17056274	A/G	0.01	0.658	3.66E-06	0.63	ZNF407
estradiol	3p26	2	3	1595644–1598393	rs402675	T/A	0.50	-0.097	6.27E-06	0.23	
estradiol	6q16	3	3	96524696–96528092	rs815653	G/T	0.05	0.210	9.69E-06	0.06	
estradiol	1q41	1	-	213021343	rs10495024	T/C	0.36	-0.096	9.83E-06	0.83	
estradiol	1q42	1	-	231200737	rs17829302	G/T	0.08	0.215	9.94E-06	0.43	
testosterone	1p36	7	9	22314053–22323369	rs909814	C/T	0.39	0.103	9.06E-07	0.44	
testosterone	4q35	1	-	191016319	rs11132733	C/T	0.17	-0.225	3.31E-06	0.59	
testosterone	17p12	4	1	12576879–12578327	rs9905820	T/G	0.42	-0.094	3.80E-06	0.41	MYOCD
testosterone	1q41	1	-	213021343	rs10495024	T/C	0.36	-0.103	5.59E-06	0.67	
testosterone	1p33	2	1015	46996943–48011680	rs12059860	T/C	0.01	0.619	8.25E-06	0.46	CYP4B1
Testosterone	20p13	1	-	4164864	rs4815670	G/A	0.44	-0.102	9.79E-06	0.13	ADRA1D

a From NCI genome build 35. ^b^Major/minor allele. ^c^Minor allele frequency.

d Combined effect sizes and P values are calculated using a fixed-effects meta-analysis (METAL software).

### Estradiol

No SNP association with estradiol levels reached the nominal genome-wide significance threshold of P = 5×10^–8^ in the meta-analysis of the NHS non-PMH users and SIBS participants (**Table 2** **, Figure S5**). The SNP most significantly associated with estradiol levels (rs6016142, P = 6.47×10^–8^), located on chromosome 20q12, is ∼632 kb downstream of the nearest gene: DEAH (Asp-Glu-Ala-His) box polypeptide 35 (*DHX35*, Gene ID: 60625). Suggestive associations were also observed for 34 SNPs in the region containing the cytochrome P450, family 19, subfamily A, polypeptide 1 (*CYP19A1*) gene on chromosome 15q21.1 (smallest joint P = 5.11×10^–7^ for rs727479; **Tables 2** **and S3**), including variants which were previously shown to be associated with estradiol levels [20], [22], [25]. Between-study heterogeneity in effect estimates ranged from very low to high (I^2^ = 0% for rs17703883 to I^2^ = 79% for rs2899472, P_heterogeneity_ >0.03) for suggestive SNPs at this locus. Genome-wide significance was not observed for any SNP association with estradiol levels among NHS women who were on PMH at blood draw (P≥4.85×10^–7^). In a secondary meta-analysis including all three groups of women, rs727479 at the *CYP19A1* locus displayed the most significant association with estradiol levels (joint P = 3.33×10^–7^, I^2^ = 4%, P_heterogeneity_ = 0.35; **Table S4**).

### Testosterone

No SNP association with testosterone levels reached genome-wide significance in the meta-analysis of NHS non-PMH users and SIBS study participants (**Tables 2** **and S5, Figure S6**). The most significant association with testosterone levels was observed for rs909814 on chromosome 1p36.12. The SNP is located ∼104 kb upstream of the nearest gene: wingless-type MMTV integration site family, member 4 (*WNT4*, Gene ID: 54361). Among the suggestive SNPs, an association was found in a region containing the cytochrome P450, family 4, subfamily B, polypeptide 1 (*CYP4B1*, Gene ID: 1580, rs12059860; joint P = 8.25x10^–6^, P_heterogeneity_ = 0.46) gene, a member of the superfamily involved in drug and steroid hormone metabolism. Additionally, the C allele of rs10495024, located ∼44 kb downstream of the estrogen-related receptor gamma (*ESRRG*, Gene ID: 2104) gene, was associated with lower levels of both estradiol (β = −0.096, joint P = 9.83×10^–6^) and testosterone (β = −0.103, joint P = 5.59×10^–6^; **Table 2**). In contrast to the SHBG and estradiol meta-analyses, none of the suggestive SNPs from the primary meta-analysis had suggestive associations with testosterone levels in the secondary meta-analysis that included the NHS PMH user group. The most significant SNP association in the meta-analysis including all three groups was observed for rs3218501, which lies within intron 2 of the X-ray repair complementing defective repair in Chinese hamster cells 2 (*XRCC2*, Gene ID: 7516) gene on chromosome 7q36.1 (joint P = 4.49×10^–7^; **Table S6**).

## Discussion

Known common genetic variants explain a small percentage of inherited breast cancer risk and identifying additional risk loci has become increasingly difficult [27]. Since a relatively high cumulative lifetime exposure to sex steroid activity is known to increase breast cancer risk, our approach was to identify novel variants through associations with plasma levels of estradiol, testosterone, and SHBG in two well-characterized, homogeneous GWAS populations of postmenopausal women. With our combined sample size, we had >80% power to detect r^2^≥0.025 at the genome-wide significance threshold of 5×10^–8^. It is reassuring that we were able to replicate two known associations in the sex steroid metabolism pathway. We also identified a novel variant, which is in relative close proximity to an estrogen receptor-related gene, that was suggestively associated with both estradiol and testosterone levels. However, we were unable to detect any novel genome-wide significant loci that influence circulating levels of estradiol, testosterone, or SHBG.

Eight regions were identified as containing at least one SNP associated with SHBG levels at the 10^–5^ level (Table 2); the most significant SNPs from each region together account for 11.4% of the variance in age-adjusted log-SHBG levels. The most significant SNPs from the 11 regions associated with estradiol explain 6.5% of the variance in age-adjusted log-estradiol levels, and the SNPs from the 6 regions associated with testosterone explain 4.4% of the variance in age-adjusted log-testosterone levels (based on the SIBS dataset). For comparison, 20%, 22% and 2% of the age-adjusted levels of log-transformed SHBG, estradiol and testosterone respectively were explained by BMI. Estimates of the total additive heritability for levels of these hormones vary (e.g. [16], [17]), but it is clear that a substantial proportion of the heritability remains to be explained. Larger genome-wide association studies may go some way towards uncovering this missing heritability (as has been the case for traits such as adult height and age at menarche [28], [29]), whilst rare variants that are inadequately captured by the standard GWAS arrays may also have a role to play.

Our study replicated associations previously observed between polymorphisms in the region of the *SHBG* gene with circulating levels of SHBG. Genome-wide significance was observed for the association with rs727428, located ∼1.1 kb downstream of the *SHBG* gene on chromosome 17, in both the NHS non-PMH user GWAS (P = 4.08×10^–8^) and SIBS GWAS (P = 8.27×10^–10^) data sets (joint P = 2.09×10^–16^). Our results are consistent with prior investigations of polymorphisms within the *SHBG* gene region and circulating SHBG levels [18]–[21], [24]. The exponentiated regression coefficient we observed for rs727428 [T] (e^β^ = 0.88 i.e. a 12% reduction in convariate-adjusted SHBG levels per T allele) is very similar to that found among postmenopausal women from the European Prospective Investigation of Cancer-Norfolk cohort study [18].

Sixteen additional SNPs at the *SHBG* locus reached genome-wide significance in our study spanning a region of ∼110 kb comprising 11 genes, with *SHBG* being the most likely candidate. However, the putative functional variant D356N (rs6259) within *SHBG*, which was previously associated with 10% higher SHBG levels among carriers [23], was not associated with SHBG levels in our primary (joint P = 0.51) or secondary meta-analysis (joint P = 0.80). Linkage disequilibrium (LD) between rs727428 and most other SNPs at this locus is low. After adjusting analyses for rs727428, associations with the other 16 SNPs were drastically attenuated (joint P≥0.004). Among women who did not use PMH at blood draw, the most significant association with plasma SHBG that remained after adjusting for rs727428 was for rs12150660 (per T allele; joint β = 0.0636, joint P = 0.004) indicating the possibility of two functional variants at this locus. Rs12150660 is strongly correlated with rs1799941, which is situated within the *SHBG* proximal promoter and has previously been reported to be associated with increased SHBG levels (*r* ^2^ = 0.95 in 1000 Genomes CEU) [18], [21]. Outside of the *SHBG* region we saw several SNPs with suggestive evidence of association, one of which (rs12596210) is intronic within the fat mass and obesity associated (*FTO*, Gene ID: 79068) gene. As SHBG levels are known to be negatively correlated with BMI, this may be the result of residual confounding.

We did not obtain genome-wide significant associations with variants for plasma estradiol levels. Among SNPs suggestively associated with estradiol levels (P<10^–5^), several SNPs at the *CYP19A1* locus were observed in our primary meta-analysis of non-PMH users and secondary meta-analysis that included the NHS PMH users. The *CYP19A1* gene codes for aromatase, which converts the androgens androstenedione and testosterone to estrone and estradiol, respectively, and is the obligate enzyme for synthesis of steroidal estrogens [30]. Rs727479 was the *CYP19A1* locus variant most significantly associated with estradiol levels in both primary and secondary meta-analyses (joint P = 5.11×10^–7^ and 3.33×10^–7^, respectively). Our results are consistent with prior reports on the association between rs727479 and estradiol levels [20], [25]. The suggestive *CYP19A1* SNPs identified by the primary meta-analysis fall within 2 haplotype blocks toward the 3′ end of the gene. After adjusting for rs727479, associations with the remaining SNPs were drastically attenuated. Three SNPS (rs2414095, rs12592697, rs4775935) in perfect LD according to the HapMap database (*r* ^2^ = 1.0), remained nominally associated with estradiol levels (P<0.05). Interestingly, these SNPs are in strong LD with rs727479 (*r^2^* = 0.96), which may indicate that they are capturing some residual signal of an unknown causal variant imperfectly tagged by rs727479. We were not able to replicate an association between a SNP close to the follicle stimulating hormone receptor (*FSHR*, Gene ID: 2492) gene (rs10454135) and postmenopausal estradiol levels that was reported by a recent candidate-gene study [20] in either the primary (P = 0.55) or secondary meta-analysis (P = 0.75).

Our study did not identify variants associated with plasma testosterone levels with genome-wide significance. One SNP associated with suggestive significance was observed at the *CYP4B1* gene locus, which is a member of the P450 family of enzymes involved in drug and steroid hormone metabolism. We also noted that rs10495024 was suggestively associated with both estradiol and testosterone levels. *ESRRG* was one of the two closest genes to rs10495024, an orphan nuclear receptor closely related to the estrogen receptors (ER) capable of regulating ER target gene expression via similar mechanisms of action [31]. While the identification of rs10495024 by both the estradiol and testosterone meta-analyses may indicate a genuine role of this locus in sex steroid biosynthesis, it is also possible that the association with testosterone levels may simply reflect the known correlation with estradiol. In fact, all of the suggestive SNPs identified by the primary testosterone meta-analysis may represent false positives, as none remained associated at P<10^−5^ in the secondary meta-analysis, which included the NHS PMH users. A recent GWAS meta-analysis of over 8000 men identified variants at the *SHBG* locus (rs12150660, joint P = 1.2×10^–41^; and rs6258, joint P = 2.3×10^–22^) and on chromosome X at the family with sequence similarity 9, member B (*FAM9B*, Gene ID: 171483) locus (rs5934505, joint P = 1.7×10^–9^) independently associated with testosterone levels [24]. These variants were not associated with circulating testosterone levels in our study of postmenopausal women in either the primary (joint P≥0.27) or secondary meta-analysis (joint P≥0.23). To our knowledge, there have not been any convincing reports of testosterone-associated SNPs among women.

Hormone signaling plays a central role in the etiology of breast cancer. Estradiol, the most biologically active hormone in breast tissue, is believed to contribute to carcinogenesis by stimulating cell proliferation and possibly also through direct genotoxic effects of its metabolites [32]. It is unclear whether the association of testosterone levels and breast cancer risk reflects a direct effect through cell proliferation or through local tissue conversion to estrogen [33]. In epidemiologic studies, adjusting for estradiol modestly attenuates the risk associated with high levels of testosterone, suggesting independent effects [12]. High-affinity binding of SHBG to estradiol and testosterone may limit their action on target tissues. This presumably accounts for the reduced risk of breast cancer observed in most studies among women with the highest SHBG levels [13]-[15]. However, despite the proposed role of these hormones in breast carcinogenesis, we did not find associations between postmenopausal breast cancer risk among the CGEMS participants and the genome-wide significant SNPs associated with plasma SHBG or the suggestive SNPs associated with plasma estradiol and testosterone. Our results are in line with what was previously seen in a comprehensive assessment of common genetic variation in known steroid metabolism genes, including *SHBG* and *CYP19A1*, which found no significant associations of these SNPs with breast cancer risk among 6,292 predominantly postmenopausal breast cancer cases and 8,135 controls (of which CGEMS participants were a part) [26]. Whilst the effects of these variants on circulating levels of SHBG and estradiol are evidently large enough to be directly detectable, it appears that they do not change levels by a sufficient amount to have a detectable effect on breast cancer risk. For example, each C allele of our most significant SNP for estradiol levels (rs6016142) was estimated to increase estradiol levels by a factor of exp^(0^.^179)^, which would be predicted to produce an approximately 6% increase in breast cancer risk, based on the effects of estradiol levels on breast cancer risk in Key et al. [13]. Therefore, it is unlikely that, individually, other common variants that influence plasma estradiol, testosterone, or SHBG levels among postmenopausal women to the same or lower extent than that identified in our study will predict breast cancer risk.

We attempted to minimize misclassification of plasma hormone levels by including only postmenopausal women in our study. By definition, postmenopausal women do not experience cyclical changes in hormone levels due to the menstrual cycle. Hormone levels among postmenopausal women do not appear to follow a diurnal pattern [34] and a single blood sample was found to reflect long-term levels relatively well with correlations of 0.68 for estradiol, 0.88 for testosterone, and 0.92 for SHBG over a period of two to three years [35]. Our primary meta-analysis was restricted to women who were not on PMH at blood draw to exclude potential heterogeneity in SNP effects driven by exogenous hormone exposure. Effect estimates observed for SNPs in the *SHBG* and *CYP19A1* regions were weaker in the NHS PMH user group, but supported the association for many of those SNPs with plasma SHBG and estradiol levels, respectively (**Tables S2 and S4**). This suggests that at least some genetic variants responsible for hormone levels are similar in the presence or absence of exogenous hormones. By restricting our study to a single blood sample from postmenopausal women, we were not able discover genetic variants that regulate hormonal diurnal variation during the prepubertal period [36], the dramatic increase in hormone levels that occurs with puberty, the cyclical variation during the menstrual cycle, or the major changes that occur at the menopause, each of which could potentially contribute to breast cancer risk later in life. Future GWAS projects in younger females may identify novel pathways that regulate hormone levels throughout different periods of life. Such knowledge could potentially contribute toward developing strategies in the prevention or treatment of this hormonally mediated disease.

## Materials and Methods

### Ethics Statement

All participants gave informed written consent. This study was approved by the Committee on Use of Human Subjects of the Brigham and Women’s Hospital, Boston, MA (NSH) as well as the Eastern Multicentre Research Ethics Committee (SIBS).

### Nurses’ Health Study Population

The Nurses’ Health Study (NHS) is a prospective cohort study of 121,700 female registered nurses in 11 states in the United States who were 30-55 years of age at enrollment. In 1976 and biennially thereafter, self-administered questionnaires were used to gather detailed information on lifestyle factors, menstrual and reproductive factors, as well as medical history. During 1989-90, blood samples were collected from 32,826 women forming a subcohort from which breast cancer cases and matched controls were selected. Eligible cases consisted of postmenopausal women with pathologically confirmed incident invasive breast cancer diagnosed anytime after blood collection up to June 1, 2004 with no prior diagnosis of cancer. Controls were randomly selected postmenopausal women free of cancer up to and including the questionnaire cycle in which the case was diagnosed. Controls were matched to cases according to age, blood collection variables [time of day, season, and year of blood collection, as well as recent (<3 months) use of postmenopausal hormones (PMH)], and ethnicity (all cases and controls are of self-reported European ancestry). Participants were defined as postmenopausal if they reported having a natural menopause or bilateral oophorectomy. Women who reported a hysterectomy with either one or both ovaries remaining were defined as postmenopausal when they were 56 years old (if a nonsmoker) or 54 years old (if a current smoker), ages at which natural menopause had occurred in 90% of the cohort. Age at menopause in the NHS is reported with a high degree of reproducibility and accuracy [37]. Completion of the self-administered questionnaire and submission of the blood sample was considered to imply informed consent.

#### Genotyping

As part of the National Cancer Institute Cancer Genetic Markers of Susceptibility (CGEMS) initiative 1,145 postmenopausal invasive breast cancer cases and 1,142 matched controls selected from the Nurses’ Health Study (NHS) were successfully genotyped using the Illumina HumanHap500 Infinium Assay (San Diego, CA) in the first stage of a three-stage GWAS of breast cancer susceptibility. Quality control metrics included removal of samples with call rates under 90% and SNP assays with call rates under 95%. Single nucleotide polymorphisms (SNP) with a minor allele frequency (MAF) of <1% were removed for a total of 528,252 genotyped SNPs [38]. Of the participants included in the CGEMS initiative, 851 cases and 852 controls had estradiol, testosterone, and/or SHBG levels measured. Principal components of genetic variation were calculated with EIGENSTRAT software [39] as described by Hunter et al., 2007 [38].

#### Hormone measurements

Details of the laboratory methods used to measure hormone levels among NHS participants are described elsewhere [40], [41]. Briefly, plasma levels of each hormone were assayed in up to 8 batches. The first 7 batches of estradiol and testosterone were measured at the Quest Diagnostics’ Nichols Institute (San Juan Capistrano, CA) using radioimmunoassay, after organic extraction and celite column chromatography. The 8^th^ batch of estradiol and testosterone was measured at the Mayo Clinic (Rochester, MN) using liquid chromatography-tandem mass spectrometry (ThermoFisher Scientific, Franklin, MA and Applied Biosystems-MDS Sciex, Foster City, CA). For SHBG, the first two batches were assayed at the University of Massachusetts Medical Center’s Longcope Steroid Radioimmunoassay Laboratory (Worchester, MA) using an immunoradiometric kit from FARMOS Diagnostica (Orion, Corp., Turku, Finland). All subsequent batches were assayed at the Reproductive Endocrinology Unit Laboratory at Massachusettes General Hospital (Boston, MA) using the AxSYM Immunoassay System (Abbott Diagnostics).

The detection limits of the radioimmunoassays were as follows: 2 pg/ml for estradiol, 2 ng/dl for testosterone, and 6.25 nmol/L for SHBG. When plasma hormone values were reported as less than the detection limit, we set the value to one unit less than the limit (estradiol, n = 8; testosterone, n = 10). Hormone values were log transformed to improve normality. Batch-specific outliers were identified using an extreme studentized deviate many-outlier procedure [42] and excluded from analyses. We included 10% blinded replicates in each batch to assess laboratory precision. Mean coefficients of variation (CV) were 13.9% for estradiol (range: 4.1% to 27.6%), 12.5% for testosterone (range: 6.6% to 13.9%), and 12.2% for SHBG (range: 9.3% to 21.9%).

### Sisters in Breast Screening Study Population

The Sisters in Breast Screening study (SIBS) was initially designed to map genes associated with mammographic breast density. Families were identified through the National Health Service breast screening program in the United Kingdom. Eligibility was restricted to families in which two or more female blood relatives (sisters, half sisters, first cousins, or aunt-niece) had had mammographic screening. Families whose second member could have screening within two years of the first member’s recruitment were also included. Study participants were sent a letter, blood kit, and questionnaire covering information on family information, reproductive and menstrual history, oral contraceptive use, PMH use, life-style factors, and medical history including benign breast disease and cancer history. Current height and weight were measured at general practices, and blood samples were collected by practice nurses or phlebotomists. Recruitment occurred between 2002 and 2010.

#### Genotyping

As part of an ongoing genome-wide study, 1302 SIBS women were genotyped using the Illumina HumanCytoSNP-12 platform. SNP assays with call rates <95%, SNPs with a MAF of <1%, or Hardy-Weinberg Equilibrium P<10^-4^ were removed for a total of 255,051 genotyped SNPs. The call rate was >99% for all samples. Six women were excluded due to less than 90% estimated European ancestry. For 9 pairs of monozygotic twins (confirmed by genotyping), the twin with the lower call rate was excluded.

#### Hormone measurements

A subset of 905 SIBS women who were aged over 55 years at recruitment, two or more years since their last menstrual period, and not currently using PMH at the time of blood collection were selected for hormone measurement at The Royal Marsden Hospital (London, UK), of whom 819 were also included in the GWAS (after exclusions described above). The women were from 390 separate families. Plasma estradiol concentrations were measured using an in-house radio-immunoassay (RIA) using a highly specific rabbit antiserum which had been raised against an estradiol-6-carboxymethyloxime-bovine serum albumin conjugate and estradiol-6-carboxymethyloxime-[2-^125^I] iodohistamine [43]. SHBG was measured using Immulite chemiluminescent immunoassay. Plasma testosterone levels were measured using RIA kit from DPC (Seimens). Estradiol measurements greater than 300 pmol/L were excluded (n = 12). The detection limits were 3 pmol/L for estradiol (n = 29/902) and 0.14 nmol/L for testosterone (n = 34/905), and values were replaced with these limits when they were reported as being undetectable. For estradiol at a concentration of 25 pmol/l the within assay variation was 6.5% and the between assay variation was 16% (n = 18). For testosterone, at a concentration of 2.5nmol/l, the within assay variation was 7% and the between assay variation was 16% (n = 28). For SHBG at a concentration of 50 nmol/l the within assay variation was 5.8% and the between assay variation was 6.6% (n = 7).

### Statistical Analyses

Genotypes for more than 2.5 million SNPs were imputed separately for the NHS and SIBS studies using MACH software [44], [45] (r^2^>0.80) and data from the HapMap European CEU panel as a reference (Phase II, release 21). Exported counts of minor allele SNP “dosages” were used in subsequent association analyses.

Among the NHS participants, association analyses were stratified by recency of PMH use at blood draw to allow for discordant associations between the two groups. The “PMH user” group consisted of women who used PMH within 3 months of blood collection, whereas the “non-PMH user” group consisted of women who had never used PMH and women who had stopped PMH use more than 3 months prior to blood collection.

The NHS and SIBS studies were both analyzed using log transformed hormone levels and each SNP’s imputed genetic dosages. Although the two studies had treated hormone values below the level of detection differently (setting to the minimum detectable value in the SIBS study and to one unit less than the detectable value in the NHS study) this affected only a very small number of participants. Additive models with 1 degree of freedom were implemented in the ProbABEL software package [46]. All analyses were adjusted for age at blood collection (continuous), body mass index (BMI, continuous; kg/m^2^), laboratory batch and previous PMH use (yes/no) (except among the NHS “PMH user” group). Testosterone analyses were additionally adjusted for age at menopause (continuous) and bilateral oophorectomy (yes/no) at the time of blood collection. The SIBS SHBG analysis was also adjusted for waist:hip ratio. NHS linear regression analyses were also adjusted for disease status, and the top principal components of genetic variation chosen after excluding any admixed individuals clearly not of European descent. Given the family-based design of the SIBS study, we used the matrix of kinship coefficients between all pairs of individuals (estimated using 8236 uncorrelated SNPs) to adjust for the non-independence of relatives in a score test for the association between SNPs and a quantitative trait. This approach is also expected to avoid the effects of population stratification in most situations [47].

Primary meta-analyses were conducted between the NHS non-PMH user group and the SIBS study participants, all of whom were non-PMH users at blood draw. Meta-analyses were based on summary statistics from the two studies including a total of 1583 women of European ancestry for the estradiol analysis, 1589 women for the testosterone analysis, and 1598 for the SHBG analysis. Secondary meta-analyses included the NHS PMH user group, which added an additional 668, 875, and 898 participants to the estradiol, testosterone, and SHBG meta-analyses, respectively (**Table 1**). For each SNP, we calculated combined effect estimates based on study-specific effect sizes and standard errors using METAL software [48]. Heterogeneity estimates were calculated using Cochran’s Q and I2 statistics. Power calculations were performed using QUANTO [49].

## Supporting Information

Figure S1 Log Quantile-Quantile P-value plot of plasma SHBG levels. The observed –log10 P-values (Y-axis) of 2,586,346 SNPs from a meta-analysis of NHS non-PMH users and SIB study participants (individual analyses adjusted as described in the Materials and Methods section) for plasma SHBG levels plotted against the expected –log10 quantile (X-axis) under the null distribution. The dashed line represents imputed P values.
(PNG)

Figure S2 Log Quantile-Quantile P-value plot of plasma Estradiol levels. The observed –log10 P-values (Y-axis) of 2,586,232 SNPs from a meta-analysis of NHS non-PMH users and SIB study participants (individual analyses adjusted as described in the Materials and Methods section) for plasma estradiol levels plotted against the expected –log10 quantile (X-axis) under the null distribution. The dashed line represents imputed P values.
(PNG)

Figure S3 Log Quantile-Quantile P-value plot of plasma Testosterone levels. The observed –log10 P-values (Y-axis) of 2,586,346 SNPs from a meta-analysis of NHS non-PMH users and SIB study participants (individual analyses adjusted as described in the Materials and Methods section) for plasma testosterone levels plotted against the expected –log10 quantile (X-axis) under the null distribution. The dashed line represents imputed P values.
(PNG)

Figure S4 Manhattan plot of plasma SHBG levels. The –log10 P-values from the meta-analysis of NHS non-PMH users and SIBS study participants (individual analyses adjusted as described in the Materials and Methods section) for plasma SHBG levels plotted against chromosomal base-pair position. The chromosomes are color coded.
(PNG)

Figure S5 Manhattan plot of plasma Estradiol levels. The –log10 P-values from the meta-analysis of NHS non-PMH users and SIBS study participants (individual analyses adjusted as described in the Materials and Methods section) for plasma estradiol levels plotted against chromosomal base-pair position. The chromosomes are color coded.
(PNG)

Figure S6 Manhattan plot of plasma Testosterone levels. The –log10 P-values from the meta-analysis of NHS non-PMH users and SIBS study participants (individual analyses adjusted as described in the Materials and Methods section) for plasma testosterone levels plotted against chromosomal base-pair position. The chromosomes are color coded.
(PNG)

Table S1 SNPs associated with log SHBG levels at P<10^−5^ from a meta-analysis of the NHS GWAS and SIBS study GWAS among non-PMH users
(PDF)

Table S2 SNPs associated with log SHBG levels at P<10^−5^ from a meta-analysis of NHS GWAS (non-PMH and PMH users) and SIBS study GWAS
(PDF)

Table S3 SNPs associated with log E2 levels at P<10^−5^ from a meta-analysis of the NHS GWAS and SIBS study GWAS among non-PMH users
(PDF)

Table S4 SNPs associated with log E2 levels at P<10^−5^ from a meta-analysis of NHS GWAS (non-PMH and PMH users) and SIBS study GWAS
(PDF)

Table S5 SNPs associated with log T levels at P<10^−5^ from a meta-analysis of the NHS GWAS and SIBS study GWAS among non-PMH users
(PDF)

Table S6 SNPs associated with log T levels at P<10^−5^ from a meta-analysis of NHS GWAS (non-PMH and PMH users) and SIBS study GWAS
(PDF)
