# Supplementary figures and images for: Genome-Wide Association Study of Circulating Estradiol, Testosterone, and Sex Hormone-Binding Globulin in Postmenopausal Women
Source: PLoS One. 2012 Jun 4;7(6):e37815. doi: 10.1371/journal.pone.0037815 (PMC3366971; doi:10.1371/journal.pone.0037815)

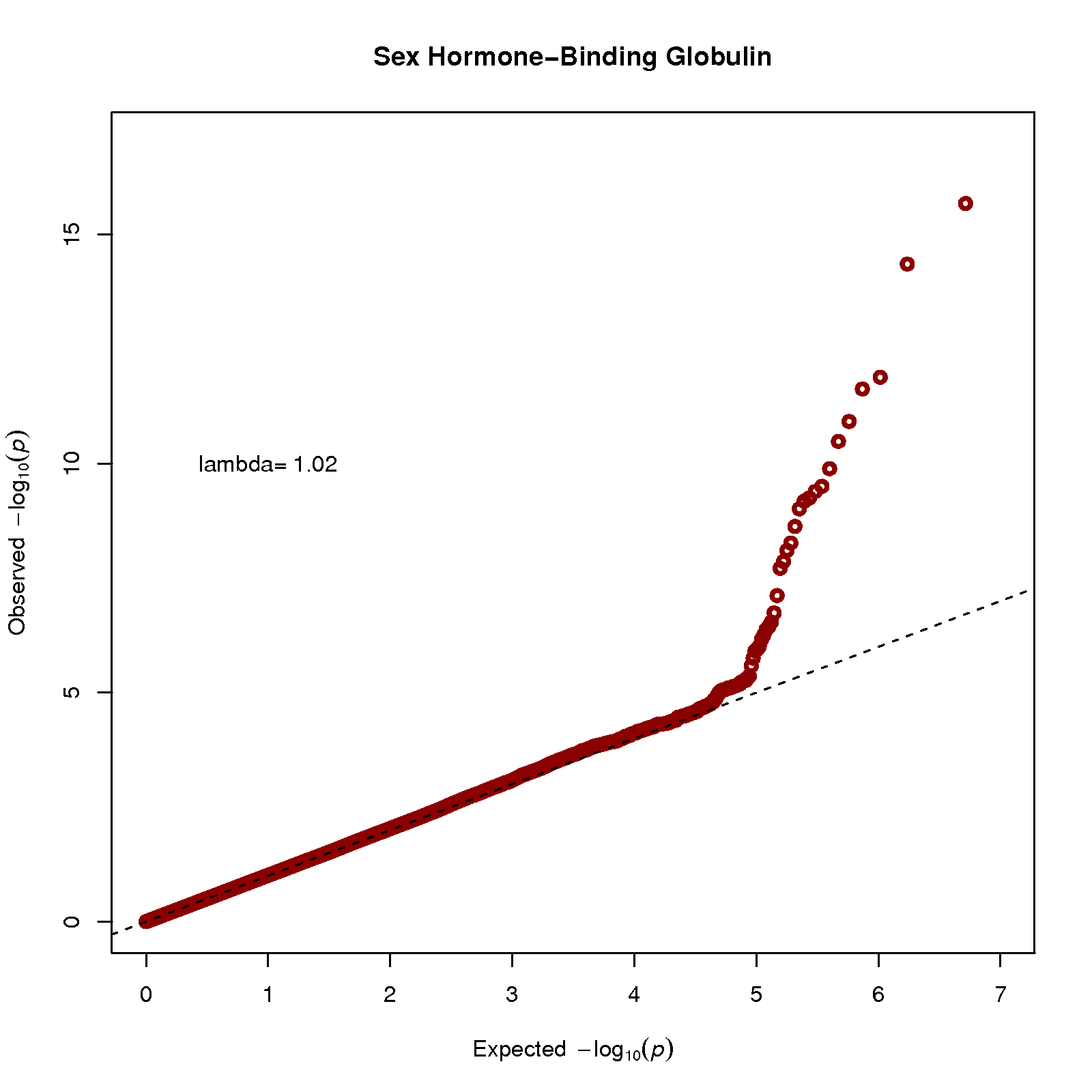

Supplement: Figure S1 — Log Quantile-Quantile P-value plot of plasma SHBG levels. The observed –log10 P-values (Y-axis) of 2,586,346 SNPs from a meta-analysis of NHS non-PMH users and SIB study participants (individual analyses adjusted as described in the Materials and Methods section) for plasma SHBG levels plotted against the expected –log10 quantile (X-axis) under the null distribution. The dashed line represents imputed P values. (PNG) [file pone.0037815.s001.png]

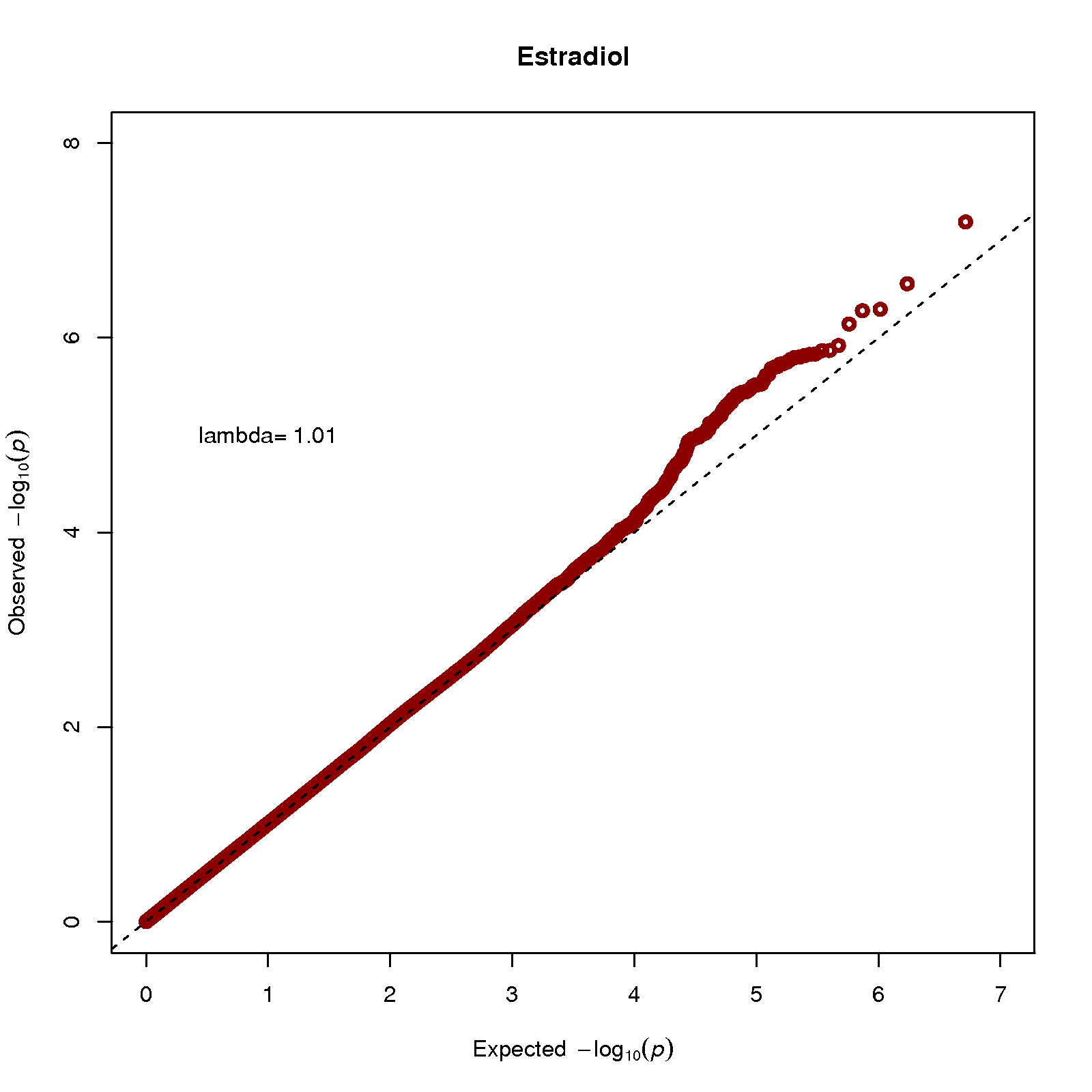

Supplement: Figure S2 — Log Quantile-Quantile P-value plot of plasma Estradiol levels. The observed –log10 P-values (Y-axis) of 2,586,232 SNPs from a meta-analysis of NHS non-PMH users and SIB study participants (individual analyses adjusted as described in the Materials and Methods section) for plasma estradiol levels plotted against the expected –log10 quantile (X-axis) under the null distribution. The dashed line represents imputed P values. (PNG) [file pone.0037815.s002.png]

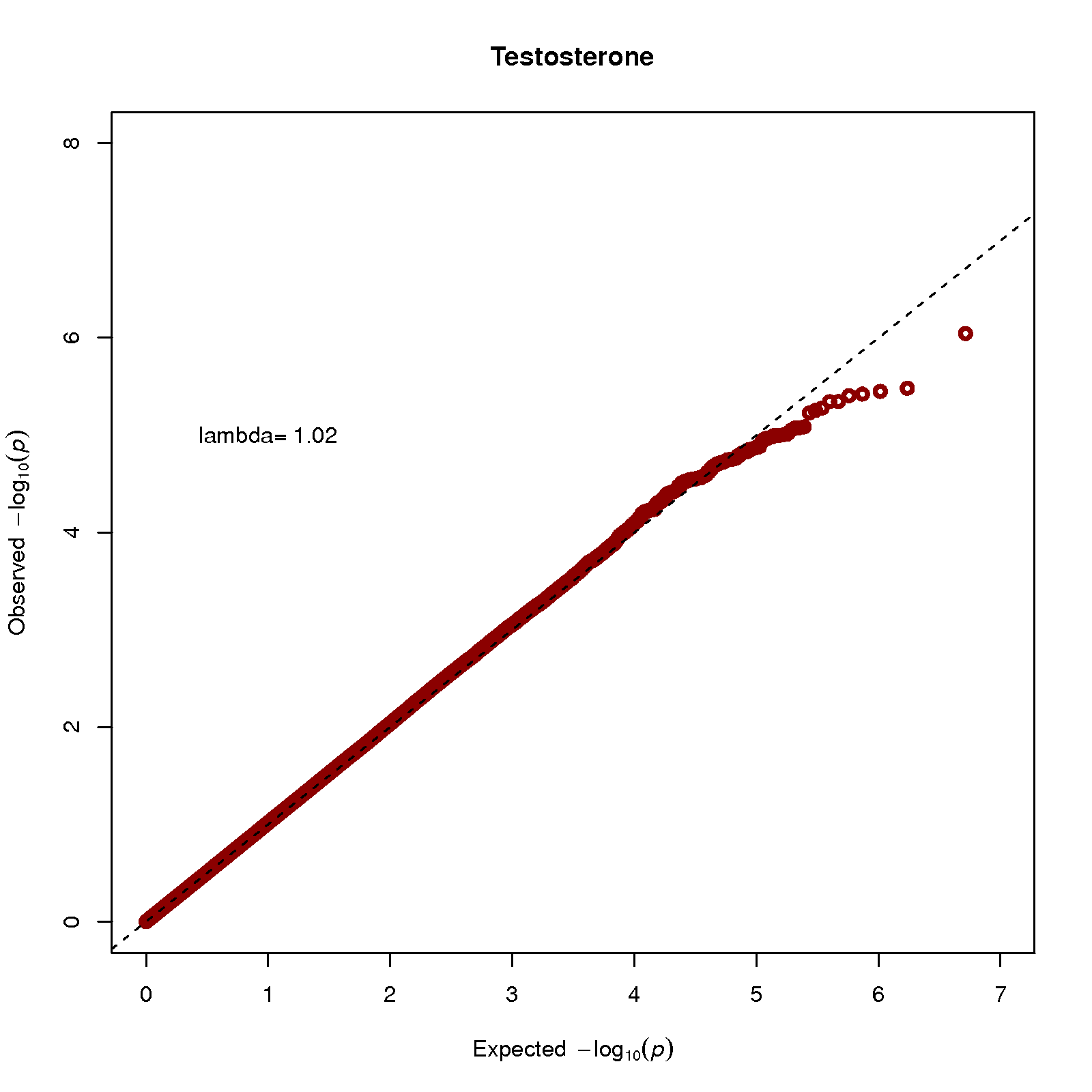

Supplement: Figure S3 — Log Quantile-Quantile P-value plot of plasma Testosterone levels. The observed –log10 P-values (Y-axis) of 2,586,346 SNPs from a meta-analysis of NHS non-PMH users and SIB study participants (individual analyses adjusted as described in the Materials and Methods section) for plasma testosterone levels plotted against the expected –log10 quantile (X-axis) under the null distribution. The dashed line represents imputed P values. (PNG) [file pone.0037815.s003.png]

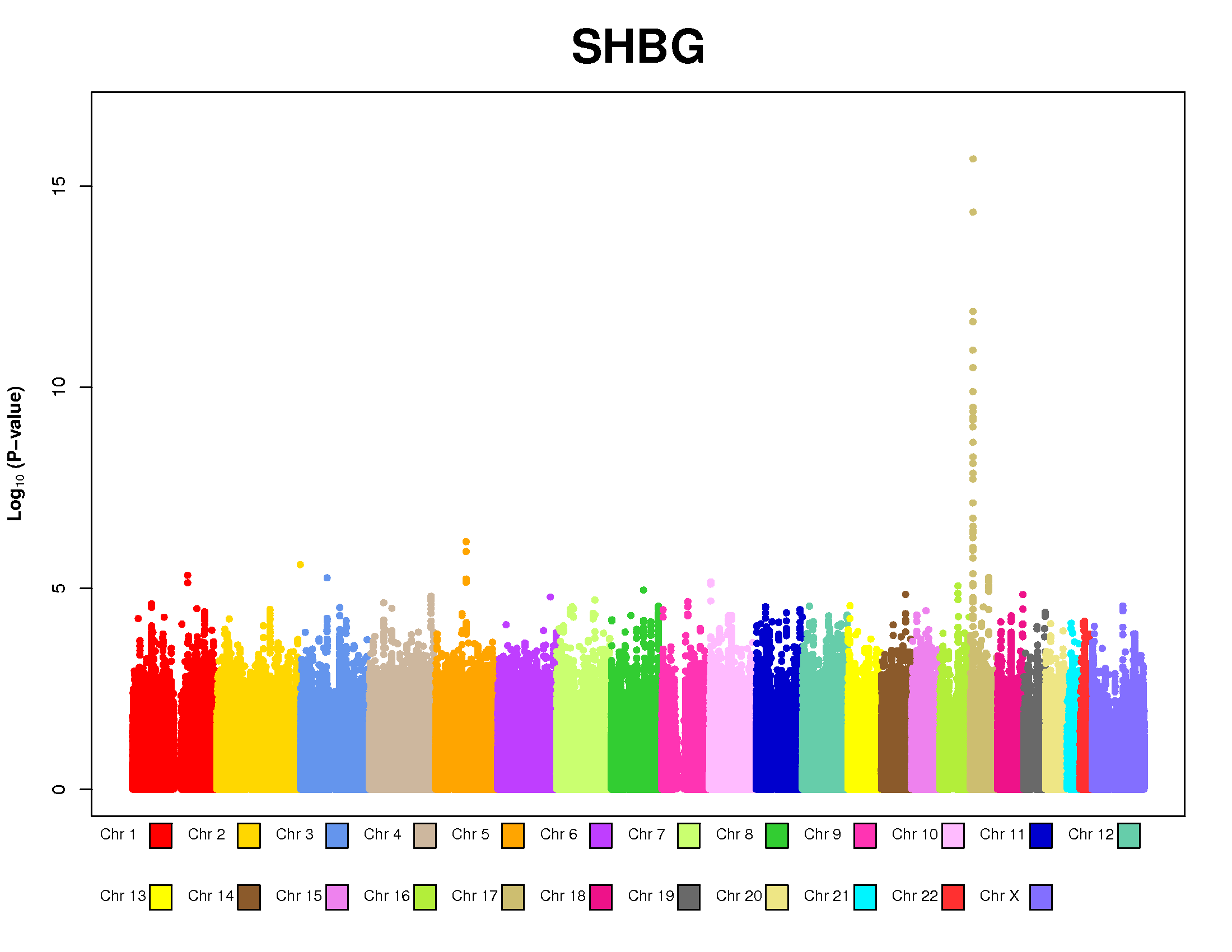

Supplement: Figure S4 — Manhattan plot of plasma SHBG levels. The –log10 P-values from the meta-analysis of NHS non-PMH users and SIBS study participants (individual analyses adjusted as described in the Materials and Methods section) for plasma SHBG levels plotted against chromosomal base-pair position. The chromosomes are color coded. (PNG) [file pone.0037815.s004.png]

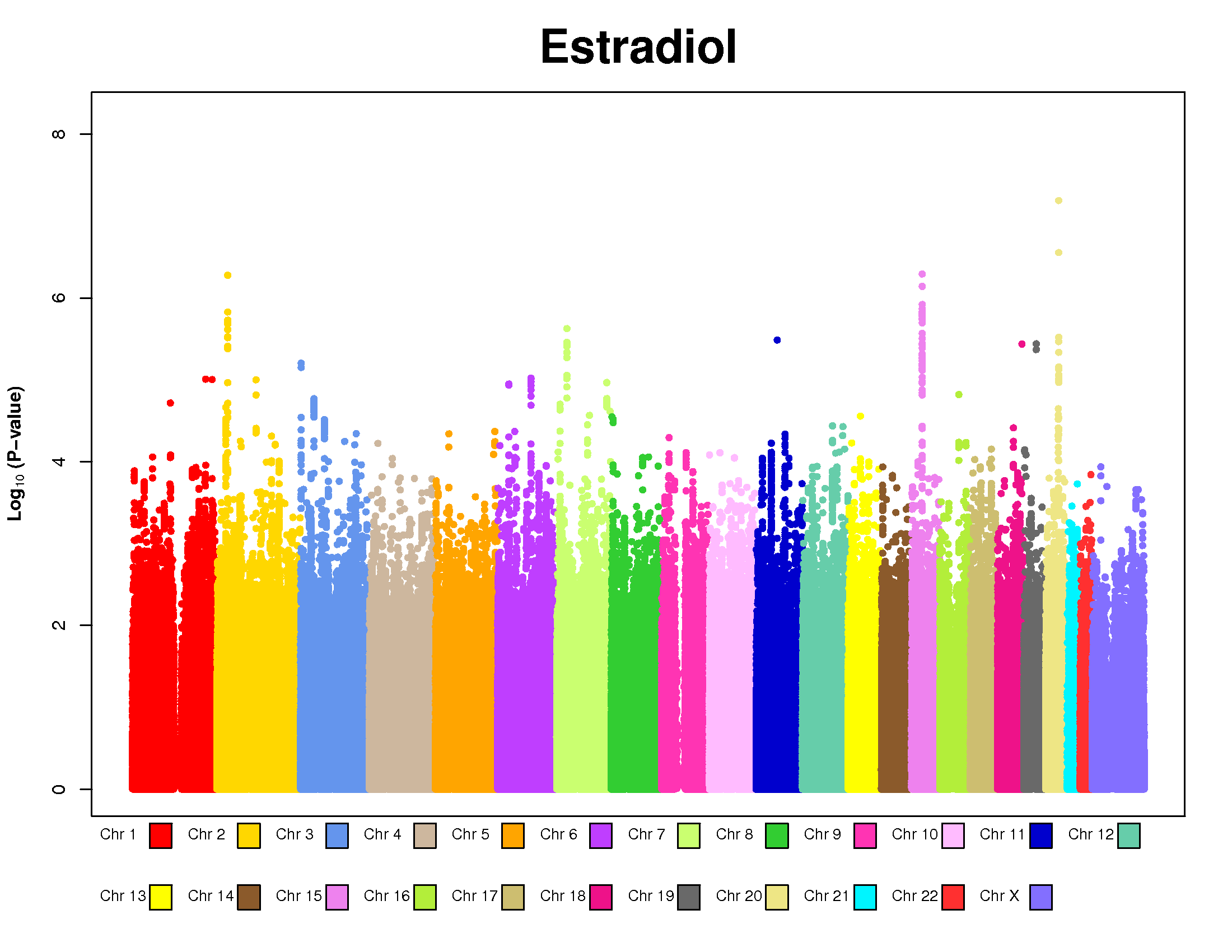

Supplement: Figure S5 — Manhattan plot of plasma Estradiol levels. The –log10 P-values from the meta-analysis of NHS non-PMH users and SIBS study participants (individual analyses adjusted as described in the Materials and Methods section) for plasma estradiol levels plotted against chromosomal base-pair position. The chromosomes are color coded. (PNG) [file pone.0037815.s005.png]

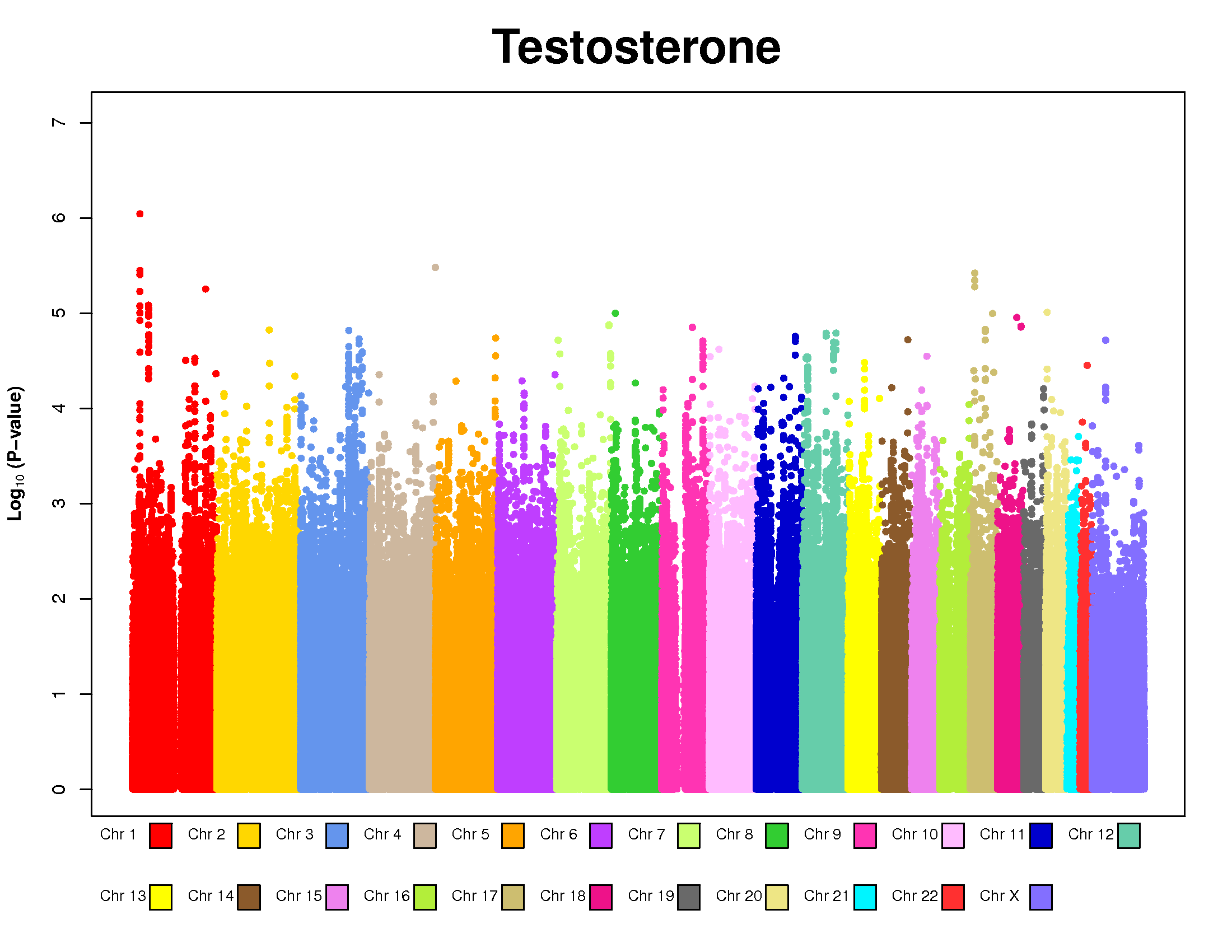

Supplement: Figure S6 — Manhattan plot of plasma Testosterone levels. The –log10 P-values from the meta-analysis of NHS non-PMH users and SIBS study participants (individual analyses adjusted as described in the Materials and Methods section) for plasma testosterone levels plotted against chromosomal base-pair position. The chromosomes are color coded. (PNG) [file pone.0037815.s006.png]
