# Supplementary material for: Genome-Wide Association Study of Circulating Estradiol, Testosterone, and Sex Hormone-Binding Globulin in Postmenopausal Women
Source: PLoS One. 2012 Jun 4;7(6):e37815. doi: 10.1371/journal.pone.0037815 (PMC3366971; doi:10.1371/journal.pone.0037815)
Supplement: Table S1 — SNPs associated with log SHBG levels at P<10−5 from a meta-analysis of the NHS GWAS and SIBS study GWAS among non-PMH users (PDF) [file pone.0037815.s007.pdf]

Table S1. SNPs associated with log SHBG levels at P < 10^{-5} from a meta-analysis of the NHS GWAS and SIBS stud

SNP	Chr	Position[a]	Gene Region (+-20kb)	WT[b]
rs727428	17	7478517	FXR2/SHBG/SAT2/ATP1B2	C
rs1641523	17	7490406	SHBG/SAT2/ATP1B2	T
rs9902027	17	7381708	TNFSF12	T
rs8073177	17	7381308	TNFSF12	C
rs12150660	17	7462640	FXR2/SHBG/SAT2	G
rs4227	17	7431901	SENP3/EIF4A1/CD68/MPDU1/SOX15/FXR2	T
rs3933469	17	7440227	EIF4A1/CD68/MPDU1/SOX15/FXR2/SHBG	G
rs2955617	17	7479510	SHBG/SAT2/ATP1B2	A
rs4602096	17	7414181	TNFSF12/TNFSF13/SENP3/EIF4A1/CD68/MPDU1/SOX15	A
rs1641537	17	7486446	SHBG/SAT2/ATP1B2	C
rs1641536	17	7486709	SHBG/SAT2/ATP1B2	C
rs1641525	17	7489936	SHBG/SAT2/ATP1B2	T
rs1619016	17	7491278	SHBG/SAT2/ATP1B2	T
rs12940684	17	7394643	TNFSF12/TNFSF13/SENP3	T
rs9899183	17	7393701	TNFSF12/TNFSF13/SENP3	T
rs4968214	17	7418201	TNFSF12/TNFSF13/SENP3/EIF4A1/CD68/MPDU1/SOX15/FXR2	G
rs4968212	17	7408940	TNFSF12/TNFSF13/SENP3/EIF4A1/CD68/MPDU1	C
rs11655920	17	7362420	POLR2A	C
rs9896688	17	7424639	TNFSF12/TNFSF13/SENP3/EIF4A1/CD68/MPDU1/SOX15/FXR2	A
rs1042522	17	7520197	ATP1B2/TP53/WRAP53	C
rs6761	17	7358387	POLR2A	T
rs2071502	17	7355682	POLR2A	C
rs4151120	17	7282872	NLGN2/SPEM1/C17orf74/TMEM102/FGF11/CHRNB1	T
rs10514317	5	89145506		C
rs9901643	17	7360548	POLR2A	G
rs3853818	17	7287026	C17orf74/TMEM102/FGF11/CHRNB1/ZBTB4	C
rs9632415	5	89070647		G
rs2241233	17	7259120	C17orf61-PLSCR3/NLGN2/SPEM1/C17orf74	T
rs6721345	2	241732719	SNED1/MTERFD2	G
rs11078685	17	7352171	POLR2A	T
rs424950	1	160688161		G
rs8077059	17	53178551		T
rs3849491	3	76566873		C
rs12941732	17	53181345		C
rs13162065	5	89138911		T
rs9303396	17	53180117		C
rs8067868	17	53179948		G
rs10795130	10	4141121		T
rs6878955	5	89125073		C
rs260918	1	160690928		G
rs10904188	10	4140191		G
rs2071504	17	7346661	ZBTB4/SLC35G6/POLR2A	C
rs7079146	10	4136692		C
rs11252345	10	4135947		G
rs13313101	10	4135640		A
rs9905773	17	53163063		A
rs9916043	17	53168259		T
rs13290	17	7270356	NLGN2/SPEM1/C17orf74/TMEM102/FGF11/CHRNB1	G
rs12596210	16	52585472	FTO	T
rs8076475	17	53169748		T
rs2058104	17	53171721		G
rs8075326	17	53168868		T

[a]From NCI genome build 35. [b]'Wildtype' or common allele. [c]'Variant' or minor allele. [d]Minor allele frequency. [e]From anal

past PMH use, laboratory batch, and four eigenvectors of the principal components identified by Eigenstrat. Shown are [illegible]
within NHS. [f]From analyses adjusting for age at blood draw, BMI at blood draw, WHR, past PMH use, and laboratory b[illegible]
[g]Combined effect sizes and P values are calculated using a fixed-effects meta-analysis (METAL software).

ly GWAS among non-PMH users

VT[c]	NHS MAF[d]	NHS β[e]	NHS P-value[e]	SIBS MAF[d]	SIBS β[f]	SIBS P-value	Joint Analysis β[g]	Joint Analysis P-value[g]	Q	I^2	$P_{heterogeneity}$[g]
T	0.40	-0.1368	4.08E-08	0.44	-0.1199	8.27E-10	-0.1263	2.09E-16	0.28	0%	0.59
C	0.38	-0.1321	3.21E-07	0.41	-0.1233	2.51E-09	-0.1267	4.40E-15	0.07	0%	0.79
C	0.23	-0.1527	1.96E-08	0.22	-0.1082	6.73E-06	-0.1277	1.30E-12	1.50	33%	0.22
T	0.23	-0.1513	4.38E-08	0.22	-0.1096	5.98E-06	-0.1277	2.35E-12	1.29	22%	0.26
T	0.26	0.1196	3.05E-05	0.26	0.1320	7.89E-08	0.1268	1.20E-11	0.11	0%	0.74
G	0.28	0.1236	6.43E-06	0.29	0.1105	1.00E-06	0.1157	3.26E-11	0.14	0%	0.71
A	0.28	0.1208	1.53E-05	0.27	0.1160	1.81E-06	0.1181	1.29E-10	0.02	0%	0.90
C	0.33	-0.1139	2.23E-06	0.35	-0.0884	2.26E-05	-0.0993	3.13E-10	0.64	0%	0.42
C	0.17	-0.1548	2.33E-07	0.17	-0.1016	1.67E-04	-0.1254	4.02E-10	1.74	43%	0.19
T	0.12	-0.1445	1.75E-05	0.13	-0.1375	7.09E-06	-0.1407	5.63E-10	0.02	0%	0.88
T	0.12	-0.1436	2.02E-05	0.12	-0.1374	7.25E-06	-0.1402	6.59E-10	0.02	0%	0.89
C	0.12	-0.1409	2.94E-05	0.12	-0.1373	7.44E-06	-0.1389	9.72E-10	0.01	0%	0.94
C	0.12	-0.1346	6.45E-05	0.12	-0.1362	8.43E-06	-0.1355	2.35E-09	0.00	0%	0.97
C	0.30	0.0931	5.80E-04	0.30	0.1079	2.09E-06	0.1019	5.41E-09	0.17	0%	0.68
C	0.27	0.0856	1.90E-03	0.27	0.1196	6.85E-07	0.1050	7.83E-09	0.85	0%	0.36
A	0.30	0.0951	3.80E-04	0.31	0.0981	8.89E-06	0.0969	1.37E-08	0.01	0%	0.93
T	0.30	0.0969	3.00E-04	0.30	0.0943	1.57E-05	0.0953	1.93E-08	0.01	0%	0.94
T	0.36	-0.0521	4.32E-02	0.40	-0.1104	1.25E-07	-0.0875	7.57E-08	3.06	67%	0.08
T	0.14	-0.1404	1.52E-05	0.13	-0.0935	1.78E-03	-0.1150	1.82E-07	1.12	11%	0.29
G	0.21	-0.1386	7.08E-06	0.20	-0.0798	3.99E-03	-0.1060	2.88E-07	2.00	50%	0.16
C	0.36	-0.0511	3.91E-02	0.40	-0.0970	1.10E-06	-0.0792	3.66E-07	2.07	52%	0.15
G	0.36	-0.0509	4.02E-02	0.40	-0.0972	1.22E-06	-0.0791	4.19E-07	2.09	52%	0.15
A	0.37	0.0644	1.14E-02	0.37	0.1458	1.68E-06	0.0982	5.55E-07	4.18	76%	0.04
T	0.13	-0.0981	4.88E-03	0.11	-0.1791	1.26E-05	-0.1323	6.96E-07	2.26	56%	0.13
A	0.13	-0.0951	3.53E-03	0.15	-0.1201	6.65E-05	-0.1087	9.69E-07	0.31	0%	0.58
T	0.42	-0.0730	2.39E-03	0.37	-0.1037	9.14E-05	-0.0870	1.14E-06	0.73	0%	0.39
A	0.06	-0.2018	6.03E-03	0.05	-0.3207	3.12E-05	-0.2589	1.23E-06	1.24	19%	0.27
C	0.15	-0.0831	8.87E-03	0.16	-0.2206	2.86E-06	-0.1265	1.77E-06	5.83	83%	0.02
A	0.01	0.7564	1.00E-01	0.01	1.2666	6.54E-06	1.1299	2.60E-06	0.88	0%	0.35
C	0.37	-0.0378	1.27E-01	0.42	-0.0930	3.05E-06	-0.0716	4.37E-06	2.97	66%	0.08
C	0.48	0.0901	1.90E-04	0.48	0.0640	5.30E-03	0.0763	4.76E-06	0.61	0%	0.43
C	0.24	-0.0974	4.10E-04	0.25	-0.0699	2.81E-03	-0.0813	5.40E-06	0.58	0%	0.45
T	0.49	-0.0716	4.25E-03	0.47	-0.0745	3.87E-04	-0.0733	5.52E-06	0.01	0%	0.93
G	0.23	-0.1041	2.00E-04	0.25	-0.0669	4.86E-03	-0.0824	5.64E-06	1.02	2%	0.31
C	0.26	-0.0537	5.12E-02	0.25	-0.1019	1.68E-05	-0.0816	5.92E-06	1.75	43%	0.19
T	0.23	-0.1009	3.00E-04	0.25	-0.0677	4.14E-03	-0.0814	6.57E-06	0.82	0%	0.36
A	0.23	-0.1006	3.20E-04	0.25	-0.0677	4.06E-03	-0.0813	6.75E-06	0.80	0%	0.37
G	0.13	0.0825	2.48E-02	0.13	0.1158	7.25E-05	0.1031	6.97E-06	0.50	0%	0.48
G	0.26	-0.0532	5.34E-02	0.25	-0.1004	2.03E-05	-0.0807	7.14E-06	1.68	40%	0.20
A	0.48	0.0884	2.00E-04	0.48	0.0601	7.53E-03	0.0734	7.37E-06	0.74	0%	0.39
A	0.13	0.0839	2.16E-02	0.14	0.1075	9.96E-05	0.0990	7.44E-06	0.26	0%	0.61
T	0.14	-0.0850	9.07E-03	0.15	-0.1065	2.34E-04	-0.0971	7.73E-06	0.24	0%	0.62
T	0.13	0.0834	2.24E-02	0.14	0.1069	1.02E-04	0.0985	7.89E-06	0.26	0%	0.61
A	0.13	0.0833	2.25E-02	0.14	0.1071	1.02E-04	0.0986	7.89E-06	0.27	0%	0.60
G	0.13	0.0831	2.27E-02	0.14	0.1072	1.01E-04	0.0985	7.97E-06	0.27	0%	0.60
T	0.25	-0.0897	1.27E-03	0.25	-0.0731	1.75E-03	-0.0799	8.40E-06	0.21	0%	0.65
C	0.23	-0.0980	4.50E-04	0.24	-0.0657	3.66E-03	-0.0784	8.53E-06	0.80	0%	0.37
T	0.36	-0.0650	1.06E-02	0.30	-0.1208	9.33E-05	-0.0877	8.71E-06	1.93	48%	0.16
C	0.10	-0.1418	5.30E-04	0.13	-0.1034	3.77E-03	-0.1199	8.74E-06	0.50	0%	0.48
C	0.23	-0.0981	4.40E-04	0.24	-0.0651	3.83E-03	-0.0781	8.84E-06	0.84	0%	0.36
A	0.23	-0.0971	5.10E-04	0.25	-0.0662	3.62E-03	-0.0784	9.11E-06	0.73	0%	0.39
G	0.23	-0.0981	4.40E-04	0.24	-0.0643	4.02E-03	-0.0774	9.49E-06	0.89	0%	0.35

lyses adjusting for age at blood draw, BMI at blood draw, case-control status,

} estimates for the minor allele variant and P-values among the non-PMH users
atch.
