# Supplementary material for: Genome-Wide Association Study of Circulating Estradiol, Testosterone, and Sex Hormone-Binding Globulin in Postmenopausal Women
Source: PLoS One. 2012 Jun 4;7(6):e37815. doi: 10.1371/journal.pone.0037815 (PMC3366971; doi:10.1371/journal.pone.0037815)
Supplement: Table S2 — SNPs associated with log SHBG levels at P<10−5 from a meta-analysis of NHS GWAS (non-PMH and PMH users) and SIBS study GWAS (PDF) [file pone.0037815.s008.pdf]

Table S2. SNPs associated with log SHBG levels at P < 10^{-5} from a meta-analysis of NHS GWAS (non-PMH and PMH [illegible]

SNP	Chr	Position[a]	Gene Region (+-20kb)	WT[b]	VT[c]
rs727428	17	7478517	FXR2/SHBG/SAT2/ATP1B2	C	T
rs1641523	17	7490406	SHBG/SAT2/ATP1B2	T	C
rs9902027	17	7381708	TNFSF12	T	C
rs8073177	17	7381308	TNFSF12	C	T
rs4227	17	7431901	SENP3/EIF4A1/CD68/MPDU1/SOX15/FXR2	T	G
rs3933469	17	7440227	EIF4A1/CD68/MPDU1/SOX15/FXR2/SHBG	G	A
rs12150660	17	7462640	FXR2/SHBG/SAT2	G	T
rs2955617	17	7479510	SHBG/SAT2/ATP1B2	A	C
rs12940684	17	7394643	TNFSF12/TNFSF13/SENP3	T	C
rs1641537	17	7486446	SHBG/SAT2/ATP1B2	C	T
rs1641536	17	7486709	SHBG/SAT2/ATP1B2	C	T
rs4968212	17	7408940	TNFSF12/TNFSF13/SENP3/EIF4A1/CD68/MPDU1	C	T
rs4968214	17	7418201	TNFSF12/TNFSF13/SENP3/EIF4A1/CD68/MPDU1/SOX15/FXR2	G	A
rs1641525	17	7489936	SHBG/SAT2/ATP1B2	T	C
rs4602096	17	7414181	TNFSF12/TNFSF13/SENP3/EIF4A1/CD68/MPDU1/SOX15	A	C
rs1619016	17	7491278	SHBG/SAT2/ATP1B2	T	C
rs9899183	17	7393701	TNFSF12/TNFSF13/SENP3	T	C
rs1042522	17	7520197	ATP1B2/TP53/WRAP53	C	G
rs9896688	17	7424639	TNFSF12/TNFSF13/SENP3/EIF4A1/CD68/MPDU1/SOX15/FXR2	A	T
rs11655920	17	7362420	POLR2A	C	T
rs2241233	17	7259120	C17orf61-PLSCR3/NLGN2/SPEM1/C17orf74	T	C
rs1005533	20	38920524		A	G
rs7798845	7	106002419		A	C
rs17477143	7	106005642		G	A
rs6761	17	7358387	POLR2A	T	C
rs7787754	7	106003786		T	A
rs7804699	7	106003905		C	T
rs11763800	7	65967634	TYW1	T	G
rs2071502	17	7355682	POLR2A	C	G
rs6698302	1	230732022	SLC35F3/MIR4671	G	A
rs4151120	17	7282872	NLGN2/SPEM1/C17orf74/TMEM102/FGF11/CHRNB1	T	A
rs6029381	20	38914643		G	A
rs7804859	7	106004020		C	T
rs8120919	20	14285517	FLRT3/MACROD2	C	T
rs9479263	6	152623924	SYNE1	G	A
rs6016445	20	38892815		G	A
rs13894	17	7470627	FXR2/SHBG/SAT2	G	A
rs6016450	20	38916313		G	A
rs11622292	14	89136080	FOXN3	T	C
rs17776811	14	89136204	FOXN3	C	A
rs11544223	X	54354049	TSR2/FGD1	A	G
rs13290	17	7270356	NLGN2/SPEM1/C17orf74/TMEM102/FGF11/CHRNB1	G	T
rs11159913	14	89139515	FOXN3	C	T
rs6029378	20	38900594		T	C
rs5961065	X	54351325	TSR2/FGD1	T	C
rs7903436	10	3446461		G	A

[a]From NCI genome build 35. [b]'Wildtype' or common allele. [c]'Variant' or minor allele. [d]Minor allele frequency. [e]From analyses [illegible]
laboratory batch, and four eigenvectors of the principal components identified by Eigenstrat. Analyses among non-PMH user [illegible]
[f]From analyses adjusting for age at blood draw, BMI at blood draw, WHR, past PMH use, and laboratory batch.
[g]Combined effect sizes and P values are calculated using a fixed-effects meta-analysis (METAL software).

users) and SIBS study GWAS

NHS (non-PMH users)			NHS (PMH users)		SIBS			Joint Analysis			
MAF[d]	β[e]	P-value[e]	β[e]	P-value[e]	MAF[d]	β[f]	P-value	β[g]	P-value[g]	Q	I[2]
0.40	-0.1368	4.08E-08	-0.0625	2.79E-02	0.44	-0.1199	8.27E-10	-0.1120	1.31E-16	4.16	52%
0.38	-0.1321	3.21E-07	-0.0642	3.00E-02	0.41	-0.1233	2.51E-09	-0.1125	2.32E-15	3.48	43%
0.23	-0.1527	1.96E-08	-0.0529	8.43E-02	0.22	-0.1082	6.73E-06	-0.1087	2.71E-12	5.90	66%
0.23	-0.1513	4.38E-08	-0.0524	9.22E-02	0.22	-0.1096	5.98E-06	-0.1087	5.08E-12	5.61	64%
0.28	0.1236	6.43E-06	0.0591	4.26E-02	0.29	0.1105	1.00E-06	0.1009	1.67E-11	2.90	31%
0.28	0.1208	1.53E-05	0.0640	3.07E-02	0.27	0.1160	1.81E-06	0.1031	4.10E-11	2.41	17%
0.26	0.1196	3.05E-05	0.0475	1.18E-01	0.26	0.1320	7.89E-08	0.1051	4.30E-11	5.02	60%
0.33	-0.1139	2.23E-06	-0.0623	2.38E-02	0.35	-0.0884	2.26E-05	-0.0902	4.67E-11	1.99	0%
0.30	0.0931	5.80E-04	0.0742	1.04E-02	0.30	0.1079	2.09E-06	0.0945	2.69E-10	0.84	0%
0.12	-0.1445	1.75E-05	-0.0672	9.33E-02	0.13	-0.1375	7.09E-06	-0.1229	4.92E-10	2.55	22%
0.12	-0.1436	2.02E-05	-0.0673	9.30E-02	0.12	-0.1374	7.25E-06	-0.1226	5.62E-10	2.50	20%
0.30	0.0969	3.00E-04	0.0769	7.28E-03	0.30	0.0943	1.57E-05	0.0906	5.74E-10	0.31	0%
0.30	0.0951	3.80E-04	0.0733	1.06E-02	0.31	0.0981	8.89E-06	0.0907	6.38E-10	0.51	0%
0.12	-0.1409	2.94E-05	-0.0674	9.35E-02	0.12	-0.1373	7.44E-06	-0.1217	7.93E-10	2.40	17%
0.17	-0.1548	2.33E-07	-0.0466	1.98E-01	0.17	-0.1016	1.67E-04	-0.1071	1.09E-09	5.33	62%
0.12	-0.1346	6.45E-05	-0.0656	1.02E-01	0.12	-0.1362	8.43E-06	-0.1187	1.95E-09	2.28	12%
0.27	0.0856	1.90E-03	0.0524	7.66E-02	0.27	0.1196	6.85E-07	0.0907	5.12E-09	3.14	36%
0.21	-0.1386	7.08E-06	-0.0548	1.14E-01	0.20	-0.0798	3.99E-03	-0.0927	1.82E-07	3.60	44%
0.14	-0.1404	1.52E-05	-0.0382	3.29E-01	0.13	-0.0935	1.78E-03	-0.0966	5.00E-07	4.02	50%
0.36	-0.0521	4.32E-02	-0.0186	5.15E-01	0.40	-0.1104	1.25E-07	-0.0707	5.84E-07	7.42	73%
0.15	-0.0831	8.87E-03	-0.0643	8.28E-02	0.16	-0.2206	2.86E-06	-0.1056	9.84E-07	7.68	74%
0.46	-0.0227	3.37E-01	-0.1020	1.10E-04	0.43	-0.0687	4.99E-04	-0.0629	1.81E-06	5.12	61%
0.26	-0.0738	5.70E-03	-0.0711	1.84E-02	0.24	-0.0709	2.74E-03	-0.0719	2.72E-06	0.01	0%
0.24	-0.0761	4.60E-03	-0.0694	2.35E-02	0.24	-0.0694	2.86E-03	-0.0715	2.93E-06	0.04	0%
0.36	-0.0511	3.91E-02	-0.0135	6.20E-01	0.40	-0.0970	1.10E-06	-0.0632	3.03E-06	6.38	69%
0.26	-0.0737	5.16E-03	-0.0676	2.28E-02	0.24	-0.0702	2.78E-03	-0.0707	3.07E-06	0.02	0%
0.26	-0.0733	5.25E-03	-0.0676	2.24E-02	0.24	-0.0701	2.79E-03	-0.0705	3.07E-06	0.02	0%
0.02	-0.3141	1.53E-03	-0.4037	1.55E-03	0.02	-0.1774	3.67E-02	-0.2692	3.09E-06	2.48	19%
0.36	-0.0509	4.02E-02	-0.0129	6.39E-01	0.40	-0.0972	1.22E-06	-0.0630	3.62E-06	6.45	69%
0.25	-0.0323	2.38E-01	-0.0927	1.98E-03	0.32	-0.0885	2.31E-04	-0.0719	3.65E-06	3.00	33%
0.37	0.0644	1.14E-02	0.0255	3.66E-01	0.37	0.1458	1.68E-06	0.0746	3.69E-06	8.63	77%
0.46	-0.0209	3.75E-01	-0.0958	3.30E-04	0.43	-0.0696	4.45E-04	-0.0610	4.01E-06	4.71	58%
0.26	-0.0706	6.77E-03	-0.0647	2.82E-02	0.24	-0.0701	2.80E-03	-0.0688	4.81E-06	0.03	0%
0.03	-0.1574	1.52E-02	-0.1752	2.19E-02	0.03	-0.1801	1.64E-03	-0.1714	4.95E-06	0.07	0%
0.03	0.2878	2.70E-04	0.1724	5.43E-02	0.02	0.4633	1.30E-02	0.2582	5.33E-06	2.26	11%
0.46	-0.0175	5.23E-01	-0.1053	7.10E-04	0.44	-0.0932	1.47E-04	-0.0715	6.17E-06	5.79	65%
0.07	-0.1332	2.41E-03	-0.0896	6.93E-02	0.07	-0.1071	3.91E-03	-0.1109	6.85E-06	0.45	0%
0.45	-0.0171	4.67E-01	-0.0956	3.20E-04	0.43	-0.0675	5.37E-04	-0.0589	6.96E-06	5.22	62%
0.39	0.0535	2.40E-02	0.0569	3.71E-02	0.40	0.0724	8.38E-04	0.0621	7.41E-06	0.39	0%
0.39	0.0548	2.13E-02	0.0556	4.27E-02	0.40	0.0725	8.35E-04	0.0623	7.45E-06	0.38	0%
0.02	-0.1876	3.23E-02	-0.4281	1.40E-05	0.01	-0.1333	5.71E-01	-0.2830	7.95E-06	3.74	46%
0.36	-0.0650	1.06E-02	-0.0402	1.44E-01	0.30	-0.1208	9.33E-05	-0.0717	8.01E-06	3.89	49%
0.35	0.0666	6.42E-03	0.0382	1.73E-01	0.34	0.0698	6.90E-04	0.0613	8.65E-06	0.89	0%
0.46	-0.0168	5.05E-01	-0.0960	8.20E-04	0.44	-0.0856	2.14E-04	-0.0654	8.79E-06	5.52	64%
0.02	-0.1972	3.18E-02	-0.4665	1.31E-05	0.01	-0.1271	5.90E-01	-0.2975	9.40E-06	4.19	52%
0.37	0.0483	5.65E-02	0.1086	1.70E-04	0.35	0.0507	2.51E-02	0.0648	9.49E-06	3.10	36%

adjusting for age at blood draw, BMI at blood draw, case-control status,
s were additionally adjusted for past PMH use.

$P_{heterogeneity}$ [g]
0.12
0.18
0.05
0.06
0.23
0.30
0.08
0.37
0.66
0.28
0.29
0.86
0.78
0.30
0.07
0.32
0.21
0.17
0.13
0.02
0.02
0.08
1.00
0.98
0.04
0.99
0.99
0.29
0.04
0.22
0.01
0.09
0.99
0.96
0.32
0.06
0.80
0.07
0.82
0.83
0.15
0.14
0.64
0.06
0.12
0.21
