# Supplementary material for: Genome-Wide Association Study of Circulating Estradiol, Testosterone, and Sex Hormone-Binding Globulin in Postmenopausal Women
Source: PLoS One. 2012 Jun 4;7(6):e37815. doi: 10.1371/journal.pone.0037815 (PMC3366971; doi:10.1371/journal.pone.0037815)
Supplement: Table S3 — SNPs associated with log E2 levels at P<10−5 from a meta-analysis of the NHS GWAS and SIBS study GWAS among non-PMH users (PDF) [file pone.0037815.s009.pdf]

Table S3. SNPs associated with log E2 levels at $P < 10^{-5}$ from a meta-analysis of the NHS GWAS and SIBS study

						NHS			
SNP	Chr	Position[a]	Gene Region (+-20kb)	WT[b]	VT[c]	MAF[d]	β[e]	P-value[e]	MAF[d]
rs6016142	20	37734221		C	T	0.11	-0.2017	1.27E-07	0.11
rs6028593	20	37725829		T	C	0.11	-0.1892	1.10E-06	0.11
rs727479	15	49321839	CYP19A1/MIR4713	A	C	0.35	-0.0919	2.40E-04	0.34
rs597800	2	31385893	EHD3	G	C	0.14	-0.1516	1.77E-05	0.15
rs17601876	15	49341201	CYP19A1/MIR4713	A	G	0.50	0.0788	6.80E-04	0.48
rs7175531	15	49321347	CYP19A1/MIR4713	C	T	0.31	-0.0939	5.00E-04	0.30
rs12595627	15	49304392	CYP19A1	C	T	0.34	-0.0852	7.00E-04	0.34
rs2414097	15	49317127	CYP19A1/MIR4713	A	G	0.34	-0.0857	6.40E-04	0.33
rs4775935	15	49306568	CYP19A1/MIR4713	G	T	0.34	-0.0841	8.00E-04	0.34
rs644503	2	31401477	EHD3	G	C	0.13	-0.1616	3.41E-05	0.12
rs12592697	15	49312465	CYP19A1	C	T	0.34	-0.0850	7.20E-04	0.33
rs749292	15	49346023	CYP19A1	G	A	0.46	0.0740	1.64E-03	0.44
rs2414095	15	49311584	CYP19A1/MIR4713	G	A	0.34	-0.0847	7.40E-04	0.33
rs3889391	15	49345714	CYP19A1	G	A	0.46	0.0739	1.68E-03	0.44
rs8039089	15	49348620	CYP19A1	T	G	0.46	0.0741	1.61E-03	0.44
rs12050767	15	49344549	CYP19A1	T	C	0.46	0.0735	1.77E-03	0.44
rs617163	2	31400205	EHD3	C	T	0.12	-0.1559	3.92E-05	0.12
rs649509	2	31376929	EHD3	C	A	0.12	-0.1480	7.03E-05	0.13
rs4774584	15	49349299	CYP19A1	G	A	0.46	0.0742	1.59E-03	0.44
rs611076	2	31386554	EHD3	G	A	0.12	-0.1482	7.94E-05	0.13
rs10488084	7	29856261	FKBP14/PLEKHA8	A	C	0.08	0.1890	1.00E-04	0.09
rs672364	2	31391076	EHD3	G	A	0.11	-0.1572	3.35E-05	0.11
rs2414101	15	49349726	CYP19A1	A	G	0.46	0.0742	1.59E-03	0.44
rs622107	2	31393291	EHD3	T	C	0.13	-0.1450	9.43E-05	0.13
rs6028607	20	37746719		A	C	0.12	-0.1643	4.49E-06	0.13
rs671403	2	31390885	EHD3	G	A	0.11	-0.1614	5.01E-05	0.10
rs654091	2	31389240	EHD3	G	A	0.12	-0.1485	7.87E-05	0.12
rs11632903	15	49351633	CYP19A1	C	T	0.46	0.0742	1.59E-03	0.44
rs2727261	11	61468707	BEST1/FTH1	C	T	0.11	0.1532	5.91E-05	0.08
rs17793544	20	37755632		T	C	0.12	-0.1641	4.52E-06	0.13
rs10254969	7	29880035	PLEKHA8	G	A	0.08	0.1880	1.70E-04	0.09
rs10519299	15	49338638	CYP19A1/MIR4713	C	G	0.46	0.0710	2.84E-03	0.44
rs10256675	7	29864911	PLEKHA8	C	T	0.08	0.1880	1.70E-04	0.09
rs11880316	19	36602969		C	A	0.01	0.4288	1.29E-05	0.01
rs17056274	18	70916034	ZNF407	A	G	0.01	0.6741	3.68E-06	0.01
rs17703883	15	49317389	CYP19A1/MIR4713	T	C	0.25	-0.0960	5.10E-04	0.25
rs10263852	7	29882593	PLEKHA8	G	A	0.08	0.1870	1.90E-04	0.09
rs669292	2	31396983	EHD3	T	C	0.13	-0.1395	1.40E-04	0.13
rs6493494	15	49337127	CYP19A1/MIR4713	G	A	0.45	0.0706	3.08E-03	0.44
rs590557	2	31373046	EHD3	G	A	0.12	-0.1419	1.40E-04	0.13
rs16965610	19	36585958		A	C	0.01	0.4241	1.54E-05	0.01
rs6016162	20	37799365		G	A	0.11	-0.1751	6.35E-06	0.11
rs10231351	7	29835797	FKBP14/PLEKHA8	A	G	0.08	0.1864	2.00E-04	0.09
rs4775932	15	49285831	CYP19A1	G	A	0.46	-0.0670	6.66E-03	0.45
rs12901187	15	49336829	CYP19A1/MIR4713	G	A	0.45	0.0697	3.51E-03	0.44
rs12911554	15	49330049	CYP19A1/MIR4713	T	C	0.44	-0.0687	4.31E-03	0.43
rs11636403	15	49336036	CYP19A1/MIR4713	C	T	0.49	0.0716	3.98E-03	0.47
rs10282327	7	29834043	FKBP14/PLEKHA8	A	G	0.08	0.1849	2.30E-04	0.09
rs4545755	15	49336336	CYP19A1/MIR4713	G	A	0.45	0.0691	4.05E-03	0.44
rs12148604	15	49288696	CYP19A1	T	C	0.46	-0.0659	7.16E-03	0.45
rs402675	3	1598393		T	A	0.49	-0.0820	7.70E-04	0.51
rs10046	15	49290278	CYP19A1	A	G	0.46	-0.0656	7.32E-03	0.45
rs2899472	15	49303347	CYP19A1/MIR4713	C	A	0.27	0.0832	1.22E-03	0.25

rs4774583	15	49293285	CYP19A1	C	T	0.46	-0.0657	7.06E-03	0.45
rs8029120	15	49292226	CYP19A1	G	T	0.46	-0.0654	7.29E-03	0.45
rs17800951	20	37853256		T	G	0.11	-0.1739	1.58E-05	0.12
rs437161	3	1595644		G	C	0.48	-0.0804	7.90E-04	0.50
rs6493487	15	49301021	CYP19A1	A	G	0.25	-0.0946	7.90E-04	0.24
rs6028642	20	37820001		G	T	0.11	-0.1719	9.82E-06	0.11
rs2289105	15	49294800	CYP19A1	C	T	0.46	-0.0653	7.25E-03	0.46
rs4441215	15	49344251	CYP19A1	C	G	0.40	-0.0764	1.30E-03	0.44
rs6493488	15	49301214	CYP19A1	C	G	0.40	-0.0738	3.31E-03	0.41
rs3784307	15	49293926	CYP19A1	A	G	0.47	-0.0649	7.33E-03	0.46
rs17206293	20	37835003		A	T	0.11	-0.1680	1.93E-05	0.12
rs13446281	7	29809559	SCRN1/FKBP14	C	G	0.08	0.1788	3.80E-04	0.09
rs17800079	20	37818404		T	C	0.11	-0.1707	1.08E-05	0.11
rs2304463	15	49295412	CYP19A1	C	A	0.46	-0.0647	7.98E-03	0.46
rs1068893	6	96525375		G	A	0.05	0.1716	8.90E-04	0.04
rs815656	6	96528092		T	C	0.05	0.1732	1.01E-03	0.04
rs10234140	7	29788672	SCRN1	C	A	0.08	0.1775	4.20E-04	0.09
rs815653	6	96524696		G	T	0.05	0.1717	8.80E-04	0.04
rs292858	20	37766993		A	G	0.13	-0.1524	1.42E-05	0.13
rs10495024	1	213021343		T	C	0.35	-0.0985	3.43E-05	0.37
rs17829302	1	231200737		G	T	0.08	0.1899	1.02E-03	0.07
rs6028637	20	37816039		C	T	0.11	-0.1681	1.33E-05	0.11

[a]From NCI genome build 35. [b]'Wildtype' or common allele. [c]'Variant' or minor allele. [d]Minor allele frequency. [e]From a
at blood draw, past PMH use, case-control status, laboratory batch, and four eigenvectors of the principal components
age at blood draw, BMI at blood draw, past PMH use, and laboratory batch. [g]Combined effect sizes and P values are

7 GWAS among non-PMH users

SIBS		Joint analysis				
β[f]	P-value	β[g]	P-value[g]	Q	I^2	$P_{heterogeneity}$[g]
-0.1108	9.37E-02	-0.1789	6.47E-08	1.42	30%	0.23
-0.1267	6.48E-02	-0.1740	2.79E-07	0.63	0%	0.43
-0.1466	2.73E-04	-0.1073	5.11E-07	1.33	25%	0.25
-0.1384	8.43E-03	-0.1475	5.29E-07	0.04	0%	0.84
0.1448	8.43E-05	0.0977	7.23E-07	2.29	56%	0.13
-0.1603	2.71E-04	-0.1122	1.20E-06	1.65	39%	0.20
-0.1411	2.42E-04	-0.1021	1.35E-06	1.47	32%	0.23
-0.1409	2.70E-04	-0.1022	1.36E-06	1.42	30%	0.23
-0.1405	2.23E-04	-0.1013	1.47E-06	1.52	34%	0.22
-0.1450	1.27E-02	-0.1564	1.49E-06	0.06	0%	0.81
-0.1376	2.87E-04	-0.1011	1.53E-06	1.33	25%	0.25
0.1501	5.55E-05	0.0959	1.58E-06	2.97	66%	0.08
-0.1369	2.93E-04	-0.1008	1.60E-06	1.32	24%	0.25
0.1509	5.60E-05	0.0959	1.67E-06	3.02	67%	0.08
0.1497	6.61E-05	0.0957	1.78E-06	2.90	66%	0.09
0.1516	5.70E-05	0.0956	1.84E-06	3.08	68%	0.08
-0.1426	1.41E-02	-0.1519	1.87E-06	0.04	0%	0.85
-0.1402	8.24E-03	-0.1454	1.99E-06	0.01	0%	0.90
0.1493	7.88E-05	0.0953	2.01E-06	2.83	65%	0.09
-0.1363	7.48E-03	-0.1440	2.08E-06	0.03	0%	0.85
0.1656	6.31E-03	0.1797	2.37E-06	0.09	0%	0.76
-0.1306	2.10E-02	-0.1489	2.44E-06	0.15	0%	0.70
0.1479	1.15E-04	0.0945	2.71E-06	2.68	63%	0.10
-0.1333	9.23E-03	-0.1409	2.99E-06	0.03	0%	0.85
-0.0848	1.24E-01	-0.1406	3.03E-06	1.46	32%	0.23
-0.1386	1.79E-02	-0.1542	3.05E-06	0.10	0%	0.75
-0.1270	1.10E-02	-0.1407	3.07E-06	0.12	0%	0.73
0.1471	1.40E-04	0.0941	3.13E-06	2.59	61%	0.11
0.1826	1.60E-02	0.1592	3.27E-06	0.12	0%	0.73
-0.0817	1.29E-01	-0.1387	3.42E-06	1.62	38%	0.20
0.1737	6.07E-03	0.1825	3.50E-06	0.03	0%	0.86
0.1539	6.18E-05	0.0942	3.60E-06	3.36	70%	0.07
0.1700	6.22E-03	0.1809	3.61E-06	0.05	0%	0.82
0.3482	1.01E-01	0.4144	3.63E-06	0.12	0%	0.73
0.3766	5.32E-01	0.6576	3.66E-06	0.23	0%	0.63
-0.1374	1.46E-03	-0.1082	3.74E-06	0.65	0%	0.42
0.1747	6.03E-03	0.1823	3.90E-06	0.02	0%	0.88
-0.1371	8.26E-03	-0.1387	3.90E-06	0.00	0%	0.97
0.1543	6.38E-05	0.0939	4.10E-06	3.39	71%	0.07
-0.1438	9.18E-03	-0.1425	4.16E-06	0.00	0%	0.98
0.3487	9.94E-02	0.4107	4.28E-06	0.10	0%	0.75
-0.0883	1.29E-01	-0.1482	4.62E-06	1.54	35%	0.22
0.1662	6.64E-03	0.1782	4.65E-06	0.07	0%	0.80
-0.1493	3.37E-05	-0.0936	4.83E-06	3.54	72%	0.06
0.1547	6.57E-05	0.0934	4.96E-06	3.47	71%	0.06
-0.1594	4.35E-05	-0.0940	5.01E-06	3.90	74%	0.05
0.1649	5.28E-05	0.0972	5.32E-06	3.79	74%	0.05
0.1660	6.74E-03	0.1772	5.37E-06	0.06	0%	0.81
0.1581	5.61E-05	0.0936	5.56E-06	3.73	73%	0.05
-0.1506	3.50E-05	-0.0926	5.87E-06	3.71	73%	0.05
-0.1424	1.06E-03	-0.0966	6.27E-06	1.46	32%	0.23
-0.1509	3.65E-05	-0.0923	6.36E-06	3.74	73%	0.05
0.2182	1.26E-04	0.1064	6.44E-06	4.66	79%	0.03

-0.1511	3.97E-05	-0.0920	6.64E-06	3.73	73%	0.05
-0.1511	3.85E-05	-0.0920	6.73E-06	3.76	73%	0.05
-0.1043	1.10E-01	-0.1546	6.96E-06	0.82	0%	0.36
-0.1367	1.27E-03	-0.0942	7.07E-06	1.33	25%	0.25
-0.1349	1.93E-03	-0.1067	7.22E-06	0.60	0%	0.44
-0.0861	1.40E-01	-0.1454	7.46E-06	1.50	33%	0.22
-0.1509	4.40E-05	-0.0914	7.54E-06	3.73	73%	0.05
-0.1192	1.00E-03	-0.0894	7.55E-06	0.97	0%	0.32
-0.1396	2.23E-04	-0.0941	7.61E-06	2.09	52%	0.15
-0.1528	4.02E-05	-0.0913	7.64E-06	3.91	74%	0.05
-0.1004	1.14E-01	-0.1492	8.70E-06	0.81	0%	0.37
0.1658	6.84E-03	0.1736	8.83E-06	0.03	0%	0.87
-0.0834	1.43E-01	-0.1429	8.84E-06	1.60	38%	0.21
-0.1505	5.04E-05	-0.0909	9.30E-06	3.71	73%	0.05
0.4075	5.02E-04	0.2105	9.53E-06	3.39	70%	0.07
0.3976	5.03E-04	0.2130	9.57E-06	3.17	68%	0.07
0.1658	6.88E-03	0.1728	9.69E-06	0.02	0%	0.88
0.4099	5.08E-04	0.2104	9.69E-06	3.42	71%	0.06
-0.0779	1.30E-01	-0.1286	9.82E-06	1.42	30%	0.23
-0.0857	1.07E-01	-0.0963	9.83E-06	0.05	0%	0.83
0.2739	2.04E-03	0.2151	9.94E-06	0.63	0%	0.43
-0.0845	1.40E-01	-0.1418	9.94E-06	1.46	32%	0.23

[illegible]nalyses adjusting for age at blood draw, BMI
[illegible] identified by Eigenstrat. [f]From analyses adjusting for
calculated using a fixed-effects meta-analysis (METAL software).
