# Supplementary material for: Genome-Wide Association Study of Circulating Estradiol, Testosterone, and Sex Hormone-Binding Globulin in Postmenopausal Women
Source: PLoS One. 2012 Jun 4;7(6):e37815. doi: 10.1371/journal.pone.0037815 (PMC3366971; doi:10.1371/journal.pone.0037815)
Supplement: Table S4 — SNPs associated with log E2 levels at P<10−5 from a meta-analysis of NHS GWAS (non-PMH and PMH users) and SIBS study GWAS (PDF) [file pone.0037815.s010.pdf]

Table S4. SNPs associated with log E2 levels at $P < 10^{-5}$ from a meta-analysis of NHS GWAS (non-PMH and PMH users) and SIBS study GWAS

						NHS (non-PMH users)			NHS (PMH users)		SIBS		
SNP	Chr	Position[a]	Gene Region (+-20kb)	WT[b]	VT[c]	MAF[d]	β[e]	P-value[e]	β[e]	P-value[e]	MAF[d]	β[f]	P-value
rs727479	15	49321839	CYP19A1/MIR4713	A	C	0.35	-0.0919	2.40E-04	-0.0610	2.04E-01	0.34	-0.1466	2.73E-04
rs6599224	3	38627954	SCN5A	G	C	0.15	0.0834	9.76E-03	0.1735	6.03E-03	0.16	0.1989	7.61E-05
rs6599225	3	38628281	SCN5A	G	A	0.15	0.0835	9.71E-03	0.1736	6.00E-03	0.16	0.2005	8.07E-05
rs6599226	3	38628288	SCN5A	G	A	0.15	0.0833	9.83E-03	0.1736	6.00E-03	0.16	0.2019	8.56E-05
rs12595627	15	49304392	CYP19A1	C	T	0.34	-0.0852	7.00E-04	-0.0692	1.50E-01	0.34	-0.1411	2.42E-04
rs6599223	3	38625464	SCN5A	C	T	0.16	0.0800	1.30E-02	0.1743	5.69E-03	0.16	0.1954	6.94E-05
rs6599227	3	38628550	SCN5A	A	G	0.15	0.0833	9.91E-03	0.1736	6.00E-03	0.16	0.2032	9.20E-05
rs6790718	3	38629513	SCN5A	G	A	0.15	0.0834	9.76E-03	0.1738	5.95E-03	0.16	0.2042	9.97E-05
rs6793943	3	38629697	SCN5A	C	T	0.16	0.0836	9.54E-03	0.1738	5.95E-03	0.16	0.2051	1.08E-04
rs2414097	15	49317127	CYP19A1/MIR4713	A	G	0.34	-0.0857	6.40E-04	-0.0639	1.84E-01	0.33	-0.1409	2.70E-04
rs4775935	15	49306568	CYP19A1/MIR4713	G	T	0.34	-0.0841	8.00E-04	-0.0642	1.81E-01	0.34	-0.1405	2.23E-04
rs6791081	3	38629778	SCN5A	G	A	0.16	0.0837	9.50E-03	0.1738	5.95E-03	0.16	0.2059	1.18E-04
rs12592697	15	49312465	CYP19A1	C	T	0.34	-0.0850	7.20E-04	-0.0638	1.84E-01	0.33	-0.1376	2.87E-04
rs6786119	3	38629813	SCN5A	T	C	0.16	0.0839	9.30E-03	0.1738	5.95E-03	0.16	0.2065	1.30E-04
rs2414095	15	49311584	CYP19A1/MIR4713	G	A	0.34	-0.0847	7.40E-04	-0.0638	1.84E-01	0.33	-0.1369	2.93E-04
rs9851962	3	38629975	SCN5A	A	G	0.16	0.0786	1.43E-02	0.1821	3.91E-03	0.16	0.2069	1.44E-04
rs7175531	15	49321347	CYP19A1/MIR4713	C	T	0.31	-0.0939	5.00E-04	-0.0516	3.18E-01	0.30	-0.1603	2.71E-04
rs6708535	2	113550070	IL36RN/IL1F10	T	C	0.01	0.3077	1.76E-03	0.4151	3.95E-02	0.02	0.4433	1.88E-03
rs17042795	2	113546071	IL36B/IL36RN/IL1F10	A	G	0.01	0.3195	1.46E-03	0.3864	6.89E-02	0.02	0.4702	1.37E-03
rs6016142	20	37734221		C	T	0.11	-0.2017	1.27E-07	0.0269	7.07E-01	0.11	-0.1108	9.37E-02
rs16958291	17	9296770	STX8	T	C	0.15	-0.0985	4.38E-03	-0.2207	9.20E-04	0.14	-0.1366	1.46E-02
rs17056274	18	70916034	ZNF407	A	G	0.01	0.6741	3.68E-06	0.4323	3.25E-01	0.01	0.3766	5.32E-01
rs13222543	7	99660108	PILRA/ZCWPW1/MEPCE	C	T	0.03	-0.2331	1.41E-02	-0.6789	2.29E-06	0.03	-0.1795	1.41E-01
rs8071640	17	9295229	STX8	A	G	0.14	-0.0943	5.75E-03	-0.2205	8.50E-04	0.13	-0.1367	1.31E-02
rs9543456	13	73243760	KLF12	T	C	0.34	0.1075	1.33E-03	0.2491	1.20E-04	0.32	0.0729	2.12E-01
rs17601876	15	49341201	CYP19A1/MIR4713	A	G	0.50	0.0788	6.80E-04	0.0033	9.45E-01	0.48	0.1448	8.43E-05
rs6493488	15	49301214	CYP19A1	C	G	0.40	-0.0738	3.31E-03	-0.0592	2.10E-01	0.41	-0.1396	2.23E-04
rs17042842	2	113559712	IL1F10	A	G	0.01	0.2914	3.02E-03	0.4114	4.04E-02	0.02	0.4009	3.23E-03
rs17042833	2	113558510	IL1F10	C	T	0.01	0.3000	3.22E-03	0.4114	4.04E-02	0.02	0.3999	3.25E-03
rs17042828	2	113557165	IL36RN/IL1F10	T	C	0.01	0.2907	3.03E-03	0.4114	4.04E-02	0.02	0.3987	3.29E-03
rs17042819	2	113556270	IL36RN/IL1F10	A	G	0.01	0.2907	3.03E-03	0.4114	4.04E-02	0.02	0.3974	3.32E-03
rs11896207	2	113567113	IL1F10	C	T	0.01	0.2904	3.06E-03	0.4129	3.97E-02	0.02	0.4158	3.14E-03
rs17042815	2	113556088	IL36RN/IL1F10	G	C	0.01	0.2882	3.11E-03	0.4114	4.04E-02	0.02	0.3970	3.33E-03
rs6542113	2	113575507	IL1RN	G	A	0.01	0.2904	3.06E-03	0.4114	4.04E-02	0.02	0.4238	3.24E-03
rs7944444	11	122099116	UBASH3B	A	T	0.41	0.1019	6.47E-03	0.2575	4.90E-04	0.43	0.1129	2.60E-02
rs9892348	17	9298108	STX8	T	C	0.14	-0.0928	7.08E-03	-0.2221	8.20E-04	0.13	-0.1291	2.00E-02
rs17042894	2	113580278	IL1RN	G	A	0.01	0.2904	3.06E-03	0.4127	4.03E-02	0.02	0.4278	3.31E-03
rs749292	15	49346023	CYP19A1	G	A	0.46	0.0740	1.64E-03	0.0122	7.95E-01	0.44	0.1501	5.55E-05

rs3889391	15	49345714	CYP19A1	G	A	0.46	0.0739	1.68E-03	0.0123	7.93E-01	0.44	0.1509	5.60E-05
rs11880316	19	36602969		C	A	0.01	0.4288	1.29E-05	0.0925	7.28E-01	0.01	0.3482	1.01E-01
rs8039089	15	49348620	CYP19A1	T	G	0.46	0.0741	1.61E-03	0.0122	7.96E-01	0.44	0.1497	6.61E-05
rs12050767	15	49344549	CYP19A1	T	C	0.46	0.0735	1.77E-03	0.0123	7.95E-01	0.44	0.1516	5.70E-05
rs2727261	11	61468707	BEST1/FTH1	C	T	0.11	0.1532	5.91E-05	0.0472	5.09E-01	0.08	0.1826	1.60E-02
rs16965610	19	36585958		A	C	0.01	0.4241	1.54E-05	0.1002	7.05E-01	0.01	0.3487	9.94E-02
rs1511962	12	98329577	ANKS1B	A	C	0.21	0.0980	8.50E-04	0.1499	8.76E-03	0.18	0.1377	8.14E-02
rs6028593	20	37725829		T	C	0.11	-0.1892	1.10E-06	0.0361	6.26E-01	0.11	-0.1267	6.48E-02
rs4774584	15	49349299	CYP19A1	G	A	0.46	0.0742	1.59E-03	0.0120	7.99E-01	0.44	0.1493	7.88E-05
rs9903869	17	9299953	STX8	C	T	0.14	-0.0919	8.05E-03	-0.2206	9.20E-04	0.13	-0.1235	2.55E-02
rs9530237	13	73243361	KLF12	T	G	0.20	0.0977	5.84E-03	0.2425	1.90E-04	0.19	0.0944	1.09E-01
rs11636403	15	49336036	CYP19A1/MIR4713	C	T	0.49	0.0716	3.98E-03	0.0339	4.92E-01	0.47	0.1649	5.28E-05
rs1564289	8	2131088		G	A	0.39	0.0904	4.50E-04	0.0801	1.29E-01	0.40	0.0923	2.04E-02
rs13084981	3	38621003	SCN5A	C	T	0.11	0.1213	1.17E-03	0.0948	1.77E-01	0.12	0.1886	3.21E-03
rs11057457	12	123129966	ZNF664-FAM101A	T	C	0.23	-0.0648	2.55E-02	-0.1793	1.03E-03	0.22	-0.1239	4.84E-03

[a]From NCI genome build 35. [b]'Wildtype' or common allele. [c]'Variant' or minor allele. [d]Minor allele frequency. [e]From analyses adjusting for age at blood draw, BMI at bloc
laboratory batch, and four eigenvectors of the principal components identified by Eigenstrat. Analyses among non-PMH users were additionally adjusted for past PMH u
[f]From analyses adjusting for age at blood draw, BMI at blood draw, past PMH use, and laboratory batch.
[g]Combined effect sizes and P values are calculated using a fixed-effects meta-analysis (METAL software).

Joint analysis				
β^g	P-value[g]	Q	I^2	$P_{heterogeneity}$[g]
-0.0998	3.33E-07	2.09	4%	0.35
0.1262	4.85E-07	4.38	54%	0.11
0.1263	5.19E-07	4.42	55%	0.11
0.1262	5.69E-07	4.46	55%	0.11
-0.0969	5.73E-07	1.86	0%	0.39
0.1243	6.04E-07	4.58	56%	0.10
0.1260	6.25E-07	4.49	55%	0.11
0.1261	6.63E-07	4.50	56%	0.11
0.1260	7.03E-07	4.49	55%	0.11
-0.0961	7.23E-07	1.95	0%	0.38
-0.0954	7.60E-07	2.02	1%	0.36
0.1259	7.62E-07	4.49	55%	0.11
-0.0952	8.05E-07	1.84	0%	0.40
0.1258	8.11E-07	4.47	55%	0.11
-0.0949	8.35E-07	1.81	0%	0.40
0.1234	1.26E-06	5.13	61%	0.08
-0.1023	1.30E-06	2.78	28%	0.25
0.3606	1.79E-06	0.69	0%	0.71
0.3704	1.82E-06	0.72	0%	0.70
-0.1431	1.97E-06	8.15	75%	0.02
-0.1274	2.45E-06	2.67	25%	0.26
0.6365	2.51E-06	0.47	0%	0.79
-0.3135	2.59E-06	8.40	76%	0.01
-0.1248	3.00E-06	2.92	31%	0.23
0.1243	3.06E-06	4.73	58%	0.09
0.0838	4.18E-06	5.67	65%	0.06
-0.0884	4.27E-06	2.54	21%	0.28
0.3405	4.77E-06	0.57	0%	0.75
0.3469	4.83E-06	0.46	0%	0.79
0.3395	4.86E-06	0.56	0%	0.76
0.3392	4.88E-06	0.55	0%	0.76
0.3430	4.89E-06	0.67	0%	0.72
0.3373	5.08E-06	0.58	0%	0.75
0.3441	5.22E-06	0.71	0%	0.70
0.1275	5.27E-06	3.63	45%	0.16
-0.1225	5.38E-06	2.98	33%	0.23
0.3448	5.40E-06	0.74	0%	0.69
0.0833	6.08E-06	5.62	64%	0.06

0.0832	6.38E-06	5.66	65%	0.06
0.3821	6.73E-06	1.42	0%	0.49
0.0830	6.78E-06	5.54	64%	0.06
0.0830	7.00E-06	5.70	65%	0.06
0.1386	7.32E-06	2.09	4%	0.35
0.3794	7.51E-06	1.32	0%	0.52
0.1117	7.51E-06	0.77	0%	0.68
-0.1381	7.57E-06	7.21	72%	0.03
0.0827	7.66E-06	5.46	63%	0.07
-0.1205	8.17E-06	2.92	31%	0.23
0.1231	8.50E-06	4.11	51%	0.13
0.0873	8.55E-06	5.17	61%	0.08
0.0894	8.74E-06	0.04	0%	0.98
0.1310	8.90E-06	1.14	0%	0.57
-0.0989	8.92E-06	3.83	48%	0.15

od draw, case-control status,
ıse.
