# Supplementary material for: Genome-Wide Association Study of Circulating Estradiol, Testosterone, and Sex Hormone-Binding Globulin in Postmenopausal Women
Source: PLoS One. 2012 Jun 4;7(6):e37815. doi: 10.1371/journal.pone.0037815 (PMC3366971; doi:10.1371/journal.pone.0037815)
Supplement: Table S5 — SNPs associated with log T levels at P<10−5 from a meta-analysis of the NHS GWAS and SIBS study GWAS among non-PMH users (PDF) [file pone.0037815.s011.pdf]

Table S5. SNPs associated with log T levels at P < 10-5 from a meta-analysis of the NHS GWAS and SIBS study

						NHS			
SNP	Chr	Position[a]	Gene Region (+-20kb)	WT[b]	VT[c]	MAF[d]	β[e]	P-value[e]	MAF[d]
rs909814	1	22319248		C	T	0.39	0.0897	8.50E-04	0.38
rs11132733	4	191016319		C	T	0.17	-0.2070	3.80E-04	0.17
rs2807339	1	22323369		C	T	0.26	0.1051	3.10E-04	0.29
rs9905820	17	12578096	MYOCD	T	G	0.40	-0.1080	4.86E-05	0.44
rs2744744	1	22314053		C	T	0.37	0.0801	2.90E-03	0.36
rs7225709	17	12577460	MYOCD	T	C	0.40	-0.1068	5.78E-05	0.43
rs12603345	17	12578327	MYOCD	T	C	0.40	-0.1069	6.11E-05	0.44
rs12944722	17	12576879	MYOCD	G	C	0.40	-0.1106	6.73E-05	0.43
rs10495024	1	213021343		T	C	0.35	-0.0975	1.60E-04	0.37
rs2744757	1	22323243		G	C	0.27	0.1016	5.20E-04	0.30
rs12059860	1	46996943	CYP4B1	T	C	0.01	0.6025	1.62E-05	0.01
rs2744756	1	22322503		C	T	0.38	0.0781	3.69E-03	0.36
rs2744747	1	22316768		C	T	0.25	0.1022	7.60E-04	0.26
rs12031340	1	48011680	LOC388630	T	C	0.02	0.4288	7.73E-06	0.02
rs4815670	20	4164864	ADRA1D	G	A	0.43	-0.1233	4.44E-06	0.45
rs2807337	1	22322677		C	T	0.38	0.0774	3.89E-03	0.36

[a]From NCI genome build 35. [b]'Wildtype' or common allele. [c]'Variant' or minor allele. [d]Minor allele frequency. [e]From a[illegible]
at blood draw, age at menopause, past PMH use, bilateral oophorectomy, case-control status, laboratory batch, and fo[illegible]
identified by Eigenstrat. [f]From analyses adjusting for age at blood draw, BMI at blood draw, age at menopause, bilate[illegible]
laboratory batch. [g]Combined effect sizes and P values are calculated using a fixed-effects meta-analysis (METAL soft[illegible]

GWAS among non-PMH users

SIBS		Joint Analysis				
β[f]	P-value	β[g]	P-value[g]	Q	I^2	$P_{heterogeneity}$[g]
0.1226	2.08E-04	0.1029	9.06E-07	0.59	0%	0.44
-0.2634	2.09E-03	-0.2250	3.31E-06	0.29	0%	0.59
0.1180	3.11E-03	0.1096	3.56E-06	0.07	0%	0.79
-0.0741	1.64E-02	-0.0935	3.80E-06	0.69	0%	0.41
0.1167	2.59E-04	0.0954	3.94E-06	0.76	0%	0.38
-0.0733	1.65E-02	-0.0923	4.53E-06	0.68	0%	0.41
-0.0748	1.63E-02	-0.0932	4.54E-06	0.61	0%	0.44
-0.0746	1.61E-02	-0.0945	5.29E-06	0.74	0%	0.39
-0.1197	9.56E-03	-0.1028	5.59E-06	0.18	0%	0.67
0.1188	3.07E-03	0.1076	5.92E-06	0.12	0%	0.73
1.2721	1.52E-01	0.6189	8.25E-06	0.56	0%	0.46
0.1131	4.65E-04	0.0925	8.41E-06	0.69	0%	0.41
0.1197	3.01E-03	0.1086	8.47E-06	0.12	0%	0.73
0.1871	1.29E-01	0.3370	9.00E-06	2.39	58%	0.12
-0.0444	3.19E-01	-0.1021	9.79E-06	2.30	56%	0.13
0.1115	5.33E-04	0.0915	9.94E-06	0.66	0%	0.42

nalyses adjusting for age at blood draw, BMI
ur eigenvectors of the principal components
ral oophorectomy, past PMH use, and
ware).
