# Supplementary material for: Genome-Wide Association Study of Circulating Estradiol, Testosterone, and Sex Hormone-Binding Globulin in Postmenopausal Women
Source: PLoS One. 2012 Jun 4;7(6):e37815. doi: 10.1371/journal.pone.0037815 (PMC3366971; doi:10.1371/journal.pone.0037815)
Supplement: Table S6 — SNPs associated with log T levels at P<10−5 from a meta-analysis of NHS GWAS (non-PMH and PMH users) and SIBS study GWAS (PDF) [file pone.0037815.s012.pdf]

Table S6. SNPs associated with log T levels at $P < 10^{-5}$ from a meta-analysis of NHS GWAS (non-PMH and PM

						NHS (non-PMH users)			NHS (P
SNP	Chr	Position[a]	Gene Region (+-20kb)	WT[b]	VT[c]	MAF[d]	β[e]	P-value[e]	β[e]
rs3218501	7	151789565	XRCC2	G	C	0.03	-0.1992	1.16E-02	-0.1854
rs3218504	7	151788964	XRCC2	G	A	0.03	-0.1999	1.13E-02	-0.1850
rs2013785	17	61666939	AP0H	G	A	0.46	0.0606	1.48E-02	0.0820
rs8079676	17	61667415	AP0H	G	A	0.46	0.0607	1.47E-02	0.0821
rs6933	17	61638692	AP0H	A	G	0.45	0.0607	1.56E-02	0.0867
rs7181230	15	38148033	BMF	A	G	0.33	0.0547	4.59E-02	0.0701
rs8178845	17	61648440	AP0H	A	C	0.44	0.0586	1.83E-02	0.0895
rs1671518	12	16280892	SLC15A5	C	T	0.28	0.1067	9.78E-05	0.0680
rs4372812	19	648514	FSTL3/PRSSL1/PALM	C	A	0.28	0.1236	1.30E-04	0.0929
rs1671516	12	16284632	SLC15A5	G	A	0.28	0.1065	1.00E-04	0.0659
rs11056836	12	16283812	SLC15A5	C	T	0.28	0.1066	1.00E-04	0.0659
rs9972424	15	38153143	L0C100131089/BMF	A	G	0.32	0.0546	4.60E-02	0.0674
rs678428	13	36382026	SMAD9	A	G	0.02	-0.2863	2.54E-03	-0.2662
rs2267113	22	26479958	MN1	C	T	0.06	-0.1617	4.10E-03	-0.1604
rs660122	13	36388223	SMAD9	T	C	0.02	-0.2907	2.62E-03	-0.2616
rs8178870	17	61637158	CEP112/APOH	T	A	0.43	0.0504	4.43E-02	0.0850
rs8082415	17	61635943	CEP112/APOH	T	C	0.43	0.0508	4.31E-02	0.0848
rs4072203	8	29674483		G	A	0.11	0.1345	7.20E-04	0.1469

[a]From NCI genome build 35. [b]'Wildtype' or common allele. [c]'Variant' or minor allele. [d]Minor allele frequency. [e]From a
menopause, bilateral oophorectomy, case-control status, laboratory batch, and four eigenvectors of the principal comp
were additionally adjusted for past PMH use.

[f]From analyses adjusting for age at blood draw, BMI at blood draw, age at menopause, bilateral oophorectomy, past P

[g]Combined effect sizes and P values are calculated using a fixed-effects meta-analysis (METAL software).

I users) and SIBS study GWAS

MH users)	SIBS			Joint Analysis				
P-value[e]	MAF[d]	β[f]	P-value	β[g]	P-value[g]	Q	I^2	$P_{heterogeneity}$[g]
8.28E-03	0.04	-0.2752	2.88E-04	-0.2188	4.49E-07	0.84	0%	0.66
8.49E-03	0.04	-0.2750	2.89E-04	-0.2189	4.50E-07	0.83	0%	0.66
7.00E-04	0.47	0.0754	1.14E-02	0.0726	1.43E-06	0.39	0%	0.82
7.00E-04	0.47	0.0753	1.20E-02	0.0726	1.47E-06	0.39	0%	0.82
3.70E-04	0.45	0.0666	2.58E-02	-0.0722	1.90E-06	0.59	0%	0.74
6.69E-03	0.37	0.1138	2.55E-04	0.0767	2.16E-06	2.12	5%	0.35
2.40E-04	0.45	0.0643	3.64E-02	-0.0720	2.22E-06	0.86	0%	0.65
1.55E-02	0.25	0.0554	1.34E-01	0.0807	3.62E-06	1.56	0%	0.46
3.32E-03	0.30	0.0233	7.17E-01	0.0986	4.17E-06	1.99	0%	0.37
1.92E-02	0.25	0.0562	1.28E-01	0.0799	4.48E-06	1.59	0%	0.45
1.92E-02	0.25	0.0559	1.30E-01	0.0799	4.49E-06	1.60	0%	0.45
8.92E-03	0.36	0.1199	3.84E-04	-0.0754	4.88E-06	2.39	16%	0.30
3.59E-03	0.03	-0.2823	8.68E-02	0.2767	6.74E-06	0.02	0%	0.99
1.55E-03	0.07	-0.1394	1.54E-01	-0.1582	7.67E-06	0.04	0%	0.98
4.18E-03	0.03	-0.2806	8.49E-02	0.2760	7.98E-06	0.05	0%	0.98
5.20E-04	0.43	0.0704	2.66E-02	0.0687	8.30E-06	0.96	0%	0.62
5.50E-04	0.43	0.0706	2.76E-02	-0.0688	8.61E-06	0.93	0%	0.63
3.20E-04	0.11	0.0179	7.22E-01	0.1104	9.23E-06	4.55	56%	0.10

alyses adjusting for age at blood draw, BMI at blood draw, age at
onents identified by Eigenstrat. Analyses among non-PMH users

MH use, and laboratory batch.
